# Maternal High Fat Diet in Lactation Impacts Hypothalamic Neurogenesis and Neurotrophic Development, Leading to Later Life Susceptibility to Obesity in Male but Not Female Mice

**DOI:** 10.1002/advs.202305472

**Published:** 2023-10-22

**Authors:** Yanchao Xu, Dengbao Yang, Lu Wang, Elżbieta Król, Mohsen Mazidi, Li Li, Yi Huang, Chaoqun Niu, Xue Liu, Sin Man Lam, Guanghou Shui, Alex Douglas, John R. Speakman

**Affiliations:** ^1^ Shenzhen key laboratory for metabolic health Center for Energy Metabolism and Reproduction Shenzhen Institutes of Advanced Technology Chinese Academy of Sciences Shenzhen 518055 P. R. China; ^2^ State Key Laboratory of Molecular Developmental Biology Institute of Genetics and Developmental Biology Chinese Academy of Sciences Beijing 100101 P. R. China; ^3^ Institute of Biological and Environmental Sciences University of Aberdeen Aberdeen Scotland AB24 2TZ UK; ^4^ University of Chinese Academy of Sciences Shijingshan Beijing 100049 P. R. China; ^5^ School of Pharmacy Key Laboratory of Molecular Pharmacology and Drug Evaluation Ministry of Education Yantai University Yantai 264005 P. R. China; ^6^ China medical university Shenyang 110000 P. R. China

**Keywords:** hypothalamic reprogramming, lactation, maternal high‐fat diet, neurogenesis, sex‐differential offspring obesity

## Abstract

Early life nutrition can reprogram development and exert long‐term consequences on body weight regulation. In mice, maternal high‐fat diet (HFD) during lactation predisposed male but not female offspring to diet‐induced obesity when adult. Molecular and cellular changes in the hypothalamus at important time points are examined in the early postnatal life in relation to maternal diet and demonstrated sex‐differential hypothalamic reprogramming. Maternal HFD in lactation decreased the neurotropic development of neurons formed at the embryo stage (e12.5) and impaired early postnatal neurogenesis in the hypothalamic regions of both males and females. Males show a larger increased ratio of Neuropeptide Y (NPY) to Pro‐opiomelanocortin (POMC) neurons in early postnatal neurogenesis, in response to maternal HFD, setting an obese tone for male offspring. These data provide insights into the mechanisms by which hypothalamic reprograming by early life overnutrition contributes to the sex‐dependent susceptibility to obesity in adult life in mice.

## Graphical abstract

Maternal high‐fat diet in lactation impairs the neurotropic development and early postnatal hypothalamic neurogenesis in both males and females. Males show a larger increased ratio of NPY to POMC neurons, setting an obese tone for male offspring. These data provide a novel mechanism by which hypothalamic reprogramming in early life contributes to the sex‐dependent susceptibility to obesity in adult life.

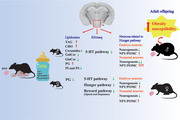

## 1 Introduction

The global prevalence of obesity represents a significant public health concern because it is an important risk factor for various chronic diseases.^[^ ^1^, ^2^, ^3^ ^]^ Obesity is characterized by disrupted energy balance resulting in excess accumulation of body fat.^[^ ^4^, ^5^, ^6^ ^]^ The incidence of obesity is primarily attributed to factors related to genetic predisposition, the environment, and their interaction.^[^ ^7^, ^8^ ^]^ Over‐consuming food with 40 to 60% fat appears to be an environmental factor in modern societies that plays a role in promoting weight gain.^[^ ^9^ ^]^

Pre‐ and post‐natal life is a developmental period with a unique sensitivity to environmental perturbations. According to the “Developmental Origins of Health and Disease” hypothesis,^[^ ^10^ ^]^ insults during early life are predicted to induce persistent alterations in the development of metabolic systems and result in elevated risk of metabolic disorders later in life. Indeed, studies in rats,^[^ ^11^, ^12^, ^13^ ^]^ mice,^[^ ^14^, ^15^, ^16^ ^]^ and humans^[^ ^17^ ^]^ have shown that maternal over‐nutrition, caused by maternal dietary fat intake during the intrauterine and/or the early postnatal period, increases the susceptibility to obesity in adulthood, particularly in an environment with a wide availability of calorie‐dense foods. Thus, maternal over‐consumption of dietary fat might be an important contributing factor to the rise in obesity rates. In rodents, the early postnatal period seems to be a more critical time window for such developmental programming.^[^ ^16^, ^18^, ^19^, ^20^, ^21^, ^22^ ^]^

The hypothalamus is a major brain region critical for body weight regulation.^[^ ^23^, ^24^ ^]^ In recent years, the hypothalamus has been extensively shown to be a critical target of developmental programming by maternal over‐nutrition.^[^ ^16^, ^25^, ^26^, ^27^, ^28^, ^29^, ^30^, ^31^, ^32^, ^33^, ^34^, ^35^, ^36^, ^37^ ^]^ In the arcuate nucleus (ARC) of the hypothalamus, there are two neuron populations that have key roles in regulating energy homeostasis: orexigenic Neuropeptide Y/Agouti‐related peptide (NPY/AgRP) neurons and anorexigenic pro‐opiomelanocortin and cocaine‐ and amphetamine‐regulated transcript (POMC/CARTPT) neurons.^[^ ^24^, ^38^, ^39^, ^40^ ^]^ In mice, neurogenesis in the ARC involving these two neuron populations mainly occurs embryonically from E10.5 to E12.5,^[^ ^41^, ^42^ ^]^ while the functional neuronal circuits linking these neurons are mainly formed in the early postnatal period.^[^ ^43^ ^]^ In fact, neurogenesis was also found in the adult hypothalamus and actively involved in the regulation of energy balance.^[^ ^44^ ^]^ There is also substantial turnover in NPY and POMC neurons in the adult hypothalamus.^[^ ^42^ ^]^ Turnover of neurons and adult neurogenesis are impaired in obese mice or by long‐term dietary fat exposure.^[^ ^42^ ^]^ The early postnatal stage during lactation may be a unique period for these important events. However, to date, very little is known about neuronal turnover and neurogenesis at this stage,^[^ ^22^ ^]^ and how they are affected by maternal dietary fat exposure. In particular, it is unclear if maternal dietary fat exposure in lactation might bias the developing system toward orexigenic and away from anorexigenic tone, thereby leading to a greater risk of obesity in later life.

In this study, we investigated the impact of maternal high‐fat diet (HFD) exclusively during lactation on the responses of both male and female offspring mice to HFD exposure during adulthood. We employed a commonly used mouse model (i.e., C57BL/6N) and standard HFD exposure (diet 45% fat by energy), and both male and female adult offspring mice were included. As in other strains,^[^ ^45^ ^]^ maternal HFD exposure during lactation exaggerated the susceptibility to obesity in male adult offspring mice but not in females. To better understand this sex difference, we selected several early postnatal timepoints during lactation to analyze the molecular (i.e., gene expression profile by RNAseq) and signatures of cellular change including neurotrophic development of the embryo‐born neurons and early postnatal neurogenesis in the hypothalamus. The effects caused by maternal HFD during lactation are mediated via changes in milk composition. The presence of microRNAs (miRNAs) and the lipid composition of milk gained much attention in recent years.^[^ ^46^, ^47^ ^]^ We investigated the detailed milk miRNAs by miRNA‐seq and lipid composition by an untargeted lipidomic approach in response to maternal HFD. Lipidomic profiles of the early postnatal offspring hypothalamus were also investigated, and these measurements together improve our understanding of the relations among diet, milk, and brain in lipid metabolism, and the mechanisms of maternal diet‐induced susceptibility to obesity in later life.

## 2 Results

### 2.1 Maternal HFD During Lactation Increases the Susceptibility to Later Life Obesity Only in Male Offspring

**Figure 1:**
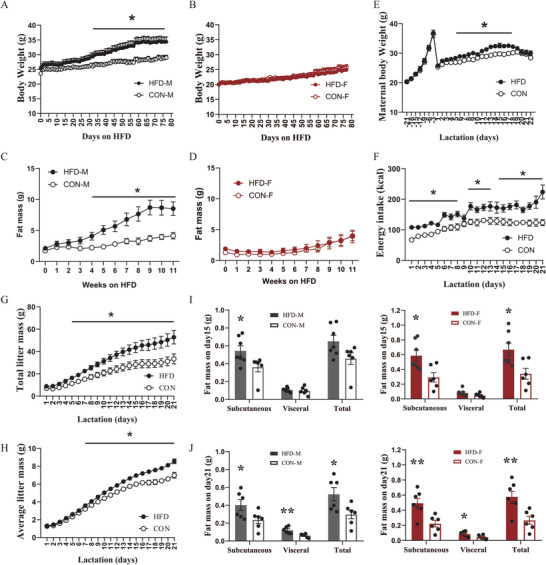
Maternal HFD feeding during lactation increases the susceptibility to HFD‐induced obesity in the male adult offspring mice, but not in females. A–D) The change of body weight (A and B) and body fat mass （C and D） in adult male (M) and female (F) offspring mice raised by mothers fed a high‐fat diet (HFD) or a control low‐fat diet (CON) during lactation in response to an 11‐week HFD exposure (male: *n =* 8; female: *n =* 9). (E and F) The change of maternal body weight and energy intake in mothers fed an HFD (*n =* 7) or a CON (*n =* 8). G,H) The effect of maternal HFD on the change of (G) total litter mass and H) average litter mass (*n =* 7–8). I,J) The effect of maternal HFD on dissected subcutaneous, visceral, and total fat mass of male and female pups on postnatal day 15 (*n =* 7) and day 21 (*n =* 6). Values are represented as mean ± SEM. Statistical tests include two‐way ANOVA (A–H) and unpaired Student's t‐test (I, J), and differences with *p <* 0.05 were considered statistically significant. *p < 0.05, **p < 0.01.

To examine the impacts of maternal HFD (45% fat by energy) during lactation on the later susceptibility of obesity development in adult offspring, both male and female offspring raised by mothers fed HFD (hereafter termed as HFD male or HFD female) or from mothers fed a low‐fat control diet (10% fat by energy) CON (termed as CON male or CON female) during lactation were exposed to HFD for 11 weeks, starting when 12 weeks old. There was a sex‐divergent response in body weight change on HFD exposure. HFD males gained much more weight (≈5 g at the end timepoint) than CON males (*p <* 0.05, **Figure** **1**A), while HFD and CON females showed no significant difference during the HFD challenge (*p >* 0.05, Figure 1B). The difference in body weight between HFD and CON males was mainly (c. 80%) due to body fat mass change (≈ 4 g at the end timepoint) (male: *p <* 0.001, female: *p >* 0.05, Figure 1C,D).

We then analyzed the details of the energy balance using metabolic chambers. Overall, male offspring showed no significant difference in energy intake and physical activity between HFD and CON group (Figure S1A,K, Supporting Information). HFD male offspring displayed positive correlations between body weight and energy intake (Figure S1C, Supporting Information) and energy expenditure (Figure S1G, Supporting Information) and maintained a relatively constant physical activity across body weight change (Figure S1M, Supporting Information). In contrast, in control male offspring there was no correlation between body weight and energy intake (Figure S1C, Supporting Information) a negative correlation between body weight and energy expenditure (Figure S1G, Supporting Information), and a positive correlation between body weight and physical activity (Figure S1M, Supporting Information). Oxygen consumption in HFD male offspring was lower compared with the control group (Figure S1E, Supporting Information). Female offspring from both groups displayed no significant difference in energy intake (Figure S1B, Supporting Information), oxygen consumption (Figure S1F, Supporting Information), physical activity (Figure S1L, Supporting Information), and similar positive correlations between body weight and energy intake (Figure S1D, Supporting Information) or energy expenditure (Figure S1G, Supporting Information), and a negative correlation between body weight and physical activity (Figure S1N, Supporting Information). In addition, HFD and CON males showed a similar level of respiratory exchange ratio (RER) (*p >* 0.05, Figure S1I, Supporting Information); while HFD females exhibited a higher RER compared to CON females (averaged 0.77 for HFD, and 0.73 for CON, *p <* 0.001, Figure S1J, Supporting Information), indicating that HFD females were able to adjust their metabolic substrate use, favoring utilization of more fat as their fuel source.

### 2.2 Maternal HFD During Lactation Increases Adiposity in the Early Postnatal Offspring

Lactating mice fed HFD had significantly higher body weight than those fed CON (Time effect: *p <* 0.0001, Group effect: *p =* 0.04, Figure 1E) and higher energy intake than those fed the CON diet (Time effect: *p <* 0.0001, Group effect: *p =* 0.0013, Figure 1F). There was no significant difference in the average litter size between HFD (litter size = 8.6) and CON group (litter size = 8.7). The litter and pup masses were significantly higher in the HFD group than those in CON group (Time effect: *p <* 0.0001, Group effect: *p <* 0.0001 for both total and average litter mass, Figure 1G,H). When the data were separated by sex, no significant difference in pup mass in both male and female offspring was observed between HFD and CON groups during lactation (*p >* 0.05, Figure S2, Supporting Information). We also dissected different fat depots from male and female pups aged day 15 and day 21. Subcutaneous (*p =* 0.04) but not visceral fat mass (*p =* 0.551) and total fat mass (*p =* 0.061) of male pups on postnatal day 15 was significantly higher in HFD group than CON group (Figure 1I). On postnatal day 21, male pups from the HFD group showed significantly higher fat mass than those from CON group in all three measures (subcutaneous fat: *p =* 0.047, visceral fat: *p =* 0.006, total fat: *p =* 0.029, Figure 1J). Similarly, in female pups, HFD group showed significantly higher fat mass than CON group on postnatal day15 and day 21 (for day 15, subcutaneous fat: *p =* 0.012, visceral fat: *p =* 0.132, total fat: *p =* 0.016; for day 21, subcutaneous fat: *p =* 0.0047, visceral fat: *p =* 0.029, total fat: *p =* 0.0061, Figure 1I,J). In summary, maternal HFD feeding during lactation increased adiposity in both male and female offspring.

### 2.3 Early Postnatal Hypothalamic Hunger Pathways are Downregulated Only in HFD Females

**Figure 2:**
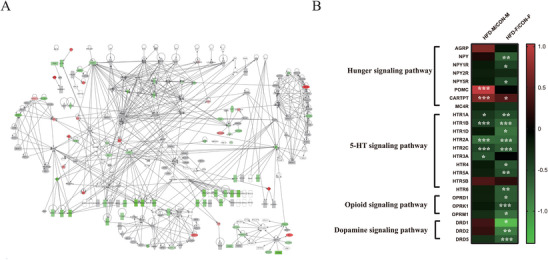
The sex‐differential effects of maternal HFD feeding during lactation on the hypothalamic transcriptome signature in early postnatal offspring mice. A) Maternal HFD feeding during lactation downregulated the key hypothalamic hunger and feeding and hedonic pathways in early postnatal offspring female mice (*n =* 5). Red indicates the upregulation, green indicates the downregulation, and gray indicates no significance. The intensity of the color is related to the absolute values of log10 (*p*‐value). B) Heatmap showing the effect of maternal HFD on the expression of genes in hunger, 5‐HT, opioid, and dopamine signaling pathways in both early postnatal male and female offspring mice. AGRP, Agouti Related Neuropeptide; NPY, Neuropeptide Y; NPY1R/2R/5R, Neuropeptide Y receptor Y1/Y2/Y5; POMC, proopiomelanocortin; CARTPT, Cocaine and amphetamine‐regulated transcript prepropeptide; MC4R, Melanocortin 4 Receptor; HTR1A/1B/1D/2A/2C/3A/4/5A/5B/6, 5‐Hydroxytryptamine Receptor 1A/1B/1D/2A/2C/3A/4/5A/5B/6; OPRD1, Opioid Receptor Delta 1; OPRK1, Opioid Receptor Kappa 1; OPRM1, Opioid Receptor Mu 1; DRD1/2/5, Dopamine Receptor D1/2/5. Values are represented as mean ± SEM and analyzed by unpaired Student's t‐test. Differences with *p <* 0.05 were considered significant. *p < 0.05, **p < 0.01, *** p< 0.001.

To examine hypothalamic gene expression, we extracted RNA from the hypothalamus of pups aged 15 days old from both HFD and CON males or females and performed bulk RNA sequencing (RNA‐seq). Ingenuity pathway analysis (IPA) focusing on pathways involved in energy balance regulation showed extensive sex‐divergent changes. Substantial downregulation of the hunger, dopamine, and opioid signaling pathways was observed in HFD females (all *p <* 0.05, **Figure** **2**A,B). The expression of Npy, Npy1r, and Npy5r (all *p <* 0.05, Figure 2A,B) was significantly reduced by maternal HFD, while there was a small but significant increase in Cartpt expression by maternal HFD (*p =* 0.014). In addition, extensive downregulation of genes in 5‐HT signaling pathway including Htr1A, Htr1B, Htr1D, Htr2A, Htr2C, Htr4, Htr5A, and Htr6 (all *p <* 0.05, Figure 2A,B) was observed in females.

In contrast, the expression of relatively few genes related to the hunger, opioid, and dopamine pathways were significantly changed by maternal HFD in males (Figure 2B). There were no significant associations between maternal HFD and the opioid or dopamine pathway (all *p >* 0.05, Figure 2B). However, Pomc and Cartpt gene expression, related to hunger inhibition, were significantly elevated by maternal HFD (all *p <* 0.01, Figure 2B). Moreover, expression of some of the serotonin (5‐HT) receptors was downregulated by maternal HFD exposure – specifically Htr1A, Htr1B, Htr2A, Htr2C, and Htr3A (all *p <* 0.05, Figure 2B). We used mathematical modeling of previously published data on the levels of different 5‐HT receptors on AgRP and POMC neurons^[^ ^48^ ^]^ to explore the potential causes of the changes in the 5‐HT receptor gene expression in both male and female offspring (**Table** **1**). These receptors are expressed differently by NPY/AgRP and POMC/CART neuron populations, so it is possible that the overall expression of these receptors by bulk RNA‐seq might stem from changed contributions of the different cell populations. The modeling data showed that the downregulation of 5‐HT receptors in hypothalamus might be caused by reduced POMC neurons under maternal HFD exposure.

**Table 1:** The trends in the RNAseq predict the changes of POMC cells under HFD feeding by equation.

Male			
Receptor	*ω*	*ψ*	*p*/*q*(*p =* 0.5)
HTR1A	0.071	0.4252	1.051
HTR1B	0.0005	0.3667	1.203
HTR1D	0.2198	0.6884	0.808
HTR2A	0.444	0.1865	1.365
HTR2C	10	0.3466	3.375
HTR4	7.355	0.6959	1.589
HTR5A	0.571	0.6286	0.582
HTR5B	0.677	2.5642	−0.044
HTR6	0.402	0.6534	0.709

Female			
Receptor	*ω*	*ψ*	*p*/*q*(*p =* 0.5)
HTR1A	0.071	0.3062	1.3098
HTR1B	0.0005	0.201	1.9145
HTR1D	0.2198	0.1668	1.9115
HTR2A	0.444	0.1932	1.3264
HTR2C	10	0.2429	4.9496
HTR4	7.355	0.2585	5.0449
HTR5A	0.571	0.3086	0.7672
HTR5B	0.677	1.6362	−0.2079
HTR6	0.402	0.2414	1.1713

*ω* The ratio of receptor gene expression observed (data from our RNAseq) in HFD and CON group.

*ψ* The ratio of receptors on AGRP and POMC cells (Data from Scott Sternsen lab)

*p* The proportion of POMC cells on CON (Assumed as *p =* 0.5)

*q* The proportion of POMC cells on HFD

p/q = 1/ψ – ^(ψ−1)^ / (ω−1) q

It is well known that the balance between the number of NPY/AGRP and POMC/CART neuron populations is critical in controlling energy balance for individuals under physiological and pathological conditions (24, 40). In addition, the balance in these two populations may contribute to the net functional changes in molecular level of appetite regulation. Since the mathematical modeling suggested the 5‐HT receptor levels might stem from differences in these neuronal populations, we next explored how maternal HFD during lactation affected the early life brain development of these neuronal populations in both male and female offspring.

### 2.4 Maternal HFD During Lactation Changes the Fate of the Embryo‐Born Neurons in Hypothalamic Regions of Early Postnatal Offspring

**Figure 3:**
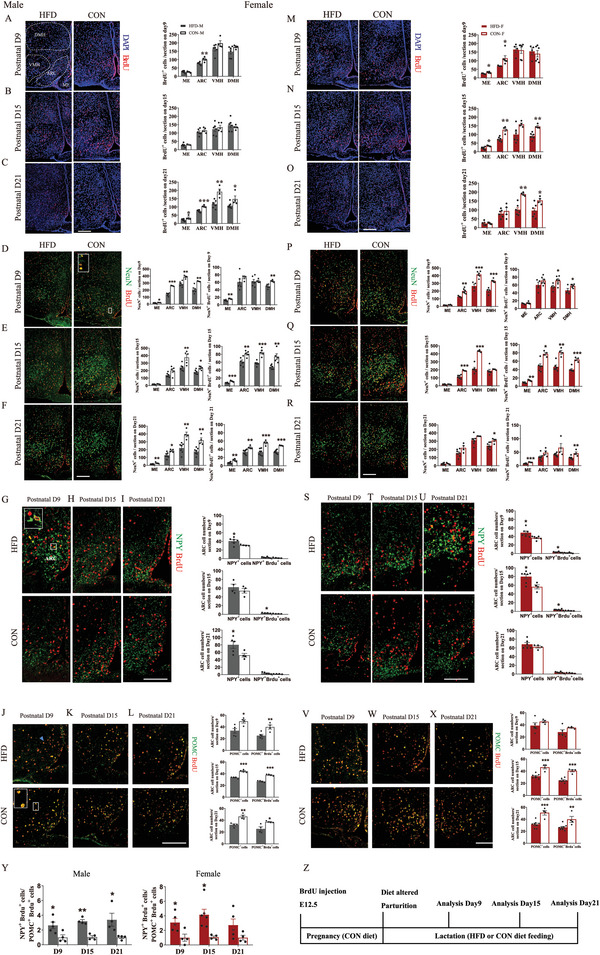
Maternal HFD feeding during lactation changes the fate of the embryo‐born cells in the hypothalamic regions of early postnatal offspring mice. A–L) Representative images (left panels) and quantification analysis (right panels) of the newborn cells labeled by BrdU on embryonic day 12.5 (E12.5) and their fate to energy balance neurons in male pups. The analysis was performed on postnatal day 9, day 15, and day 21. BrdU^+^ cells (A–C) (counterstained with DAPI) in hypothalamic regions of the male pups raised by mothers fed an HFD or a CON. Colocalization of D‐F) NeuN, G–I) NPY, and J–L) POMC with BrdU (*n =* 4–6). M–X) Representative images (left panels) and quantification analysis (right panels) of the newborn cells labeled by BrdU on E12.5 and their fate to energy balance neurons in female pups. The analysis was performed on postnatal day 9, day 15, and day 21. BrdU^+^ cells (M–O) (counterstained with DAPI) in different hypothalamic regions of the female pups raised by mothers fed an HFD or a CON. Colocalization of (P–R) NeuN, S–U) NPY, and V–X) POMC with BrdU (*n =* 4–6). Y) The ratio of BrdU^+^ cells differentiated to NPY neurons to BrdU^+^ cells differentiated to POMC neurons in both males (left) and females (right) (*n =* 4–5) Z) Schematic diagram of the experimental protocol for the analysis of embryo‐newborn neuron fate. ME, median eminence; ARC, arcuate nucleus; VMH, ventromedial nucleus; DMH, dorsomedial nucleus. Scale bars, 100 µm. Values are represented as mean ± SEM and analyzed by unpaired Student's t‐test. Differences with *p <* 0.05 were considered significant. *p < 0.05, **p < 0.01.

To investigate the impacts of maternal HFD on the fate of the embryo‐born neurons in the hypothalamus, we injected BrdU into pregnant mice at E12.5 to label newborn cells and traced their differentiation into different types of neurons by co‐labeling with NeuN, NPY, and POMC at days 9, 15, and 21 during lactation (see **Figure** **3**Z for the experimental design). In males, the BrdU^+^ cells were significantly decreased by maternal HFD in the ARC on postnatal day 9 (Median eminence ME: *p =* 0.851, ARC: *p =* 0.007, ventromedial hypothalamus VMH: *p =* 0.113, dorsomedial hypothalamus DMH: *p =* 0.150, Figure 3A) and in all hypothalamic regions on postnatal day 21 (all *p <* 0.05, Figure 3C). No significant differences were observed on postnatal day 15 (all *p >* 0.05, Figure 3B). In females, the BrdU^+^ cells were most significantly decreased by maternal HFD on postnatal day 15 (ME: *p =* 0.01, ARC: *p =* 0.04, VMH: *p =* 0.871, DMH: *p =* 0.633 for day 9; Figure 3M; ME: *p =* 0.025, ARC: *p =* 0.002, VMH: *p =* 0.055, DMH: *p =* 0.002 for day 15; Figure 3N; ME: *p =* 0.696, ARC: *p =* 0.33, VMH: *p =* 0.001, DMH: *p =* 0.04 for day 21; Figure 3O). The total numbers of neurons labeled by NeuN in nearly all hypothalamic regions were significantly decreased by maternal HFD in both males and females on postnatal day 9 (all *p <* 0.05 for males in all regions; ME: *p =* 0.0504 and all *p <* 0.05 for females in all other regions; Figure 3D,P), day 15 (ME: *p =* 0.27, ARC: *p =* 0.05, VMH: *p =* 0.008, DMH: *p =* 0.01 for males; ME: *p =* 0.563, ARC: *p =* 0.0006, VMH: *p =* 0.0002, DMH: *p =* 0.459 for females; Figure 3E,Q) and day 21 (all *p <* 0.05 for males in all regions; ME: *p =* 0.706, ARC: *p =* 0.059, VMH: *p =* 0.133, DMH: *p =* 0.016 for females; Figure 3F,R). Co‐labeling of BrdU with NeuN indicated that substantial proportion of BrdU^+^ cells were neurons (D9: 63%−67%; D15: 45%−55%; D21: 42%−52%). Similarly, the differentiation of BrdU^+^ cells into neurons labeled by NeuN in almost all hypothalamic regions was significantly decreased by maternal HFD in both males and females on postnatal day 9 (ME: *p =* 0.003, ARC: *p =* 0.249, VMH: *p =* 0.906, DMH: *p =* 0.004 for males; ME: *p =* 0.218, ARC: *p =* 0.198, VMH: *p =* 0.04, DMH: *p =* 0.03 for females; Figure 3D,P), day 15 (all *p <* 0.01 for males and females in all regions, Figure 3E,Q), and day 21 (all *p <* 0.01 for males in all regions; ME: *p <* 0.0001; ARC: *p =* 0.076; VMH: *p =* 0.051; DMH: *p =* 0.005 for females; Figure 3F,R).

We then analyzed the co‐localization of BrdU with NPY or POMC to determine the impacts of maternal HFD during lactation on the fate of BrdU^+^ cells into NPY neurons or POMC neurons in the ARC. The total numbers of NPY neurons in the ARC were significantly increased by maternal HFD on postnatal day 9 (*p =* 0.04) and day 21 (*p =* 0.04) in males (Figure 3G–I), and on postnatal day 9 (*p =* 0.02) and day 15 (*p =* 0.01) in females (Figure 3S–U). The differentiation of BrdU^+^ cells into NPY neurons was increased by maternal HFD on postnatal day 15 (*p =* 0.01) in males (Figure 3G–I) and on postnatal day 9 (*p =* 0.01) and day 15 (*p =* 0.03) in females (Figure 3S–U). The total numbers of POMC neurons were significantly decreased by maternal HFD on postnatal day 9 (*p =* 0.01), day 15 (*p =* 0.0002) and day 21 (*p =* 0.002) in males (Figure 3J–L), and on postnatal day 15 (*p =* 0.0004) and day 21 (*P =* 0.0006) in females, but not on postnatal day 9 (*p >* 0.05) (Figure 3V–X). The differentiation of BrdU^+^ cells into POMC neurons was also decreased by maternal HFD on postnatal day 9 (*p =* 0.007), day 15 (*p <* 0.0001), and day 21 (*p =* 0.017) in males, and on postnatal day 15 (*p <* 0.0001) and day 21 (*p =* 0.008) in females.

Overall, the ratios between NPY^+^BrdU^+^ and POMC^+^BrdU^+^ cells were dramatically increased by maternal HFD on postnatal day 9 (all *p <* 0.05) and day 15 (all *p <* 0.05) in both males and females and day 21 only in males (*p =* 0.03) (Figure 3Y). The retained higher ratios of the NPY to POMC neurons born during E12.5 in males may set a tone for male HFD offspring being prone to obesity development in later life.

### 2.5 Maternal HFD During Lactation Impairs Hypothalamic Neurogenesis in Early Postnatal Offspring, but to a Larger Extent in Males

We labeled the newborn cells in hypothalamus by BrdU injection into the offspring (note: most of labeled BrdU^+^ were differentiated into neurons later, see Figure 5 and so we will call this postnatal neurogenesis) to determine the impact of maternal HFD during lactation on hypothalamic neurogenesis on postnatal days 9, 15 and 21.

**Figure 4:**
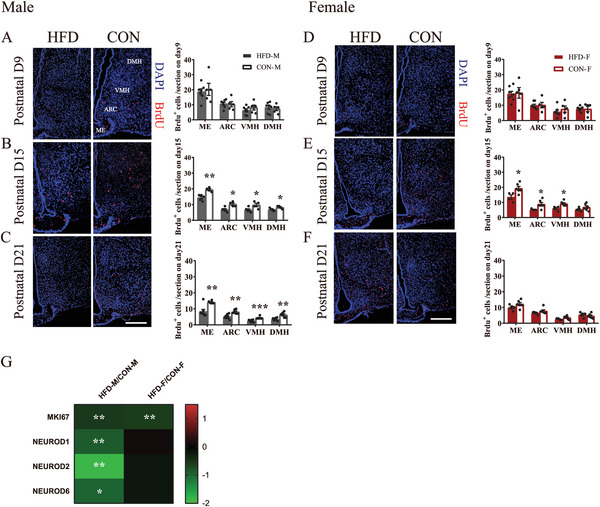
Maternal HFD feeding during lactation impairs hypothalamic neurogenesis in early postnatal offspring but lasts a longer period in males. A–C) Immunostaining (left panel) and quantification (right panel) of BrdU^+^ cells in hypothalamus of male pups on postnatal A) day 9, B) day 15, and C) day 21 (*n =* 5–7). D–F) Immunostaining (left panel) and quantification (right panel) of BrdU^+^ cells in hypothalamus of female pups on postnatal D) day 9, E) day 15, and F) day 21 (*n =* 5–7). G) The gene expression of cell proliferation maker MKI67 and neuronal differentiation markers (NEUROD1, 2 and 6) in the hypothalamus of pups on postnatal day 15 (*n =* 5) MKI67, Marker of Proliferation Ki‐67; NEUROD, Neuronal Differentiation. Scale bars, 100 µm. Values are represented as mean ± SEM and analyzed by unpaired Student's t‐test. Differences with *p <* 0.05 were considered significant. *p < 0.05, **p < 0.01.

On postnatal day 9, BrdU^+^ cells in all hypothalamic regions remained unaffected by maternal HFD in both males (all *p >* 0.05, **Figure** **4**A) and females (all *p >* 0.05, Figure 4D). On postnatal day 15, maternal HFD significantly decreased BrdU^+^ cells in most hypothalamic regions in both males (all *p <* 0.05, Figure 4B) and females (VMH: *p =* 0.252, all *p <* 0.05 for other regions, Figure 4E). On postnatal day 21, hypothalamic BrdU^+^ cells were decreased by maternal HFD only in male offspring (all *p <* 0.01, Figure 4C), but not in females (all *p >* 0.05, Figure 4F). Consistent with these observations, RNAseq data showed that Marker of Proliferation Ki‐67 (MKI67) in the hypothalamus was significantly downregulated by maternal HFD during lactation at day 15, while the gene expression of neuronal differentiation 1 (NEUROD1, *p =* 0.008), NEUROD2 (*p =* 0.002) and NEUROD6 (*p =* 0.03) were significantly downregulated by maternal HFD only in males but not in females, suggesting the possibility of more impairment of neuron differentiation in males (Figure 4G). These analyses at early postnatal timepoints indicated that hypothalamic neurogenesis was inhibited by maternal HFD during lactation in both males and females, but overall, over a longer period in males.

### 2.6 The Fate of Newborn Cells in ARC is Affected by Maternal HFD During Lactation

**Figure 5:**
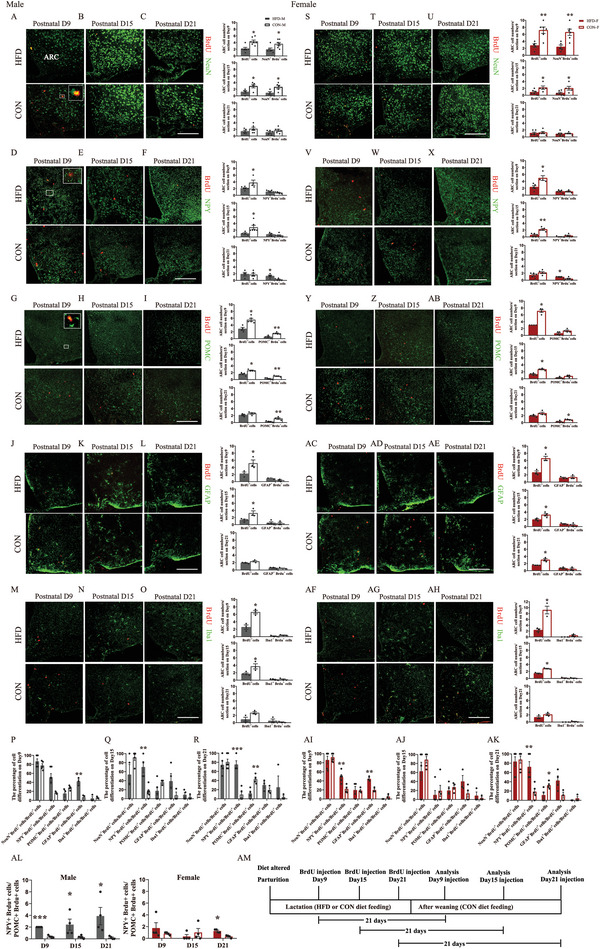
The destiny of newborn cells in hypothalamic ARC regions of early postnatal offspring is affected by maternal HFD feeding during lactation. A–O) Representative images (left panels) and quantification analysis (right panels) of the fate of the newborn cells labeled by BrdU in male pups injected with BrdU on postnatal day 9, day 15, and day 21, and the analysis was performed at 21 days after BrdU injection. Colocalization of A–C) NeuN, D–F) NPY, G–I) POMC, J–L) GFAP, M‐O) Iba1 with BrdU (*n =* 3–6). P–R) The percentage of BrdU^+^ cells differentiated to cells labeled with NeuN, NPY, POMC, GFAP, or Iba1 to total BrdU^+^ cells in male pups injected with BrdU on postnatal P) day 9, Q) day 15, and R) day 21 (*n =* 3–6). S‐AH) Representative images (left panels) and quantification analysis (right panels) of the fate of the newborn cells labeled by BrdU in female pups injected with BrdU on postnatal day 9, day 15, and day 21, and the analysis was performed at 21 days after BrdU injection. Colocalization of S–U) NeuN, V‐X) NPY, (Y‐AB) POMC, (AC–AE) GFAP, (AF–AH) Iba1 with BrdU (*n =* 3–6). AI–AK) The percentage of BrdU^+^ cells differentiated to cells labeled with NeuN, NPY, POMC, GFAP, and Iba1 to total BrdU^+^ cells in female pups injected with BrdU on postnatal (AI) day 9, (AJ) day 15, and (AK) day 21 (*n =* 3–6). AL) The ratio of BrdU^+^ cells differentiated to NPY neurons to BrdU^+^ cells differentiated to POMC neurons in both males (left) and females (right) (*n =* 4). AM) Schematic diagram of the experimental protocol for the analysis of early postnatal newborn cell fate. Scale bars, 100 µm. Values are represented as mean ± SEM and analyzed by unpaired Student's t‐test. Differences with *p <* 0.05 were considered significant. *p < 0.05, **p < 0.01.

To investigate whether maternal HFD affected the fate of these newborn cells in hypothalamic ARC related to the regulation of energy balance, we traced their differentiation into different cell populations in a separate experiment by co‐labeling analysis after weaning. Briefly, 21 days after BrdU injection in offspring on postnatal days 9, 15, and 21 (i.e., analysis on postnatal days 30, 36, and 42), we analyzed the differentiation of labeled BrdU^+^ cells by co‐labeling with three different neuronal markers (NeuN, NPY, and POMC) and two glial markers (GFAP and IBA1) (see **Figure** **5**AM for the experimental design). The number of BrdU^+^ cells in the ARC was still significantly downregulated by maternal HFD in both males and females analyzed on postnatal day 30 (male: *p =* 0.022, female: *p =* 0.003, Figure 5A,S) and postnatal day 36 (male: *p =* 0.041, female: *p =* 0.04, Figure 5B,T), but not on postnatal day 42 (male: *p =* 0.247, female: *p =* 0.739, Figure 5C,U). Most of BrdU^+^ cells were co‐labeled with NeuN (range of percentage between BrdU^+^ neurons to BrdU^+^ cells: D30: 85%−90%; D36: 50%−85%; D42: 70%−85%; Figure 5A–C and Figure 5S–U), and these percentages were not impacted by maternal HFD (all *p >* 0.05, Figure 5P–R and 5AI–K). BrdU^+^ neurons were significantly decreased by maternal HFD in both males and females on postnatal day 30 (all *p <* 0.05, Figure 5A,S) and day 36 (all *p <* 0.05, Figure 5B,T), but not on day 42 (all *p >* 0.05, Figure 5C,U). Thus, lineage tracing analysis further confirmed that neurogenesis in the ARC was impaired by maternal HFD.

We then analyzed the co‐labeling of BrdU and NPY or POMC in the ARC. The differentiation of BrdU^+^ cells into NPY neurons was not affected by maternal HFD in both males and females analyzed on postnatal day 30 (all *p >* 0.05, Figure 5D,V) and day 36 (all *p >* 0.05, Figure 5E,W), but increased by maternal HFD on day 42 (all *p <* 0.05, Figure 5F,X). The percentage of BrdU^+^NPY^+^ cells to BrdU^+^ cells was increased by maternal HFD in males analyzed on day 36 and day 42 (day 30, *p =* 0.101, day 36, *p =* 0.001, day 42, *p =* 0.0003, Figure 5P–R), and in females analyzed on postnatal day 30 and day 42 (day 30, *p =* 0.009, day 36, *p =* 0.579, day 42, *p =* 0.004, Figure 5AI–AK). Maternal HFD dramatically reduced the number of BrdU^+^ cells differentiated into POMC neurons in males analyzed on postnatal day 30 (*p =* 0.001; Figure 5G), day 36 (*p =* 0.005; Figure 5H) and day 42 (*p =* 0.009; Figure 5I), while in females, this differentiation was decreased by maternal HFD only on postnatal day 42 (*p =* 0.015; Figure 5Y‐AB). The percentage of BrdU^+^POMC^+^ cells to BrdU^+^ cells was decreased by maternal HFD in males and females analyzed on postnatal day 42 (day 30: *p =* 0.08, day 36: *p =* 0.17, day 42: *p =* 0.004 for males; day 30: *p =* 0.92, day 36: *p =* 0.477, day 42: *p =* 0.038 for females; Figure 5P–R and 5AI–AK). Overall, we found that the ratios between NPY^+^BrdU^+^ and POMC^+^BrdU^+^ cells were dramatically increased by maternal HFD in males analyzed at all these three timepoints (all *p <* 0.05, Figure 5AL‐left panel), but increased in females only on postnatal day 42 (day 30: *p =* 0.353, day 36: *p =* 0.415, day 42: *p =* 0.002; Figure 5AL‐right panel).

Differentiation of BrdU+ cells into glial cells including GFAP^+^ astrocyte and IBA^+^ microglia was not affected by maternal HFD in both males (all *p >* 0.05 for GFAP, Figure 5J–L; all *p >* 0.05 for IBA1, Figure 5M–O) and females (all *p >* 0.05 for GFAP, Figure 5AC–AE; all *p >* 0.05 for IBA1, Figure 5AF–AH) at all timepoints. The percentage of BrdU^+^GFAP^+^ to BrdU^+^ cells was only increased by maternal HFD in both males and females analyzed on postnatal day 30 (all *p <* 0.05, Figure 5P–R,AI–AK), and the percentages of BrdU^+^IBA^+^ to BrdU^+^ cells remained unaffected by maternal HFD in both males and females analyzed at all timepoints (all *p >* 0.05, Figure 5P–R,AI–AK).

In summary, we found that newborn cells in ARC between days 9 and 21, which were later differentiated mostly into neurons, were affected by maternal HFD, with overall impaired neurogenesis, elevated differentiation into NPY neurons, and decreased differentiation into POMC neurons. These differential changes led to a higher ratio of newborn NPY cells to POMC cells in both males and females when their mothers were fed HFD, but the effect was much larger in males.

### 2.7 miRNAs and Lipidomics Profiling in Response to Maternal HFD During Lactation

**Figure 6:**
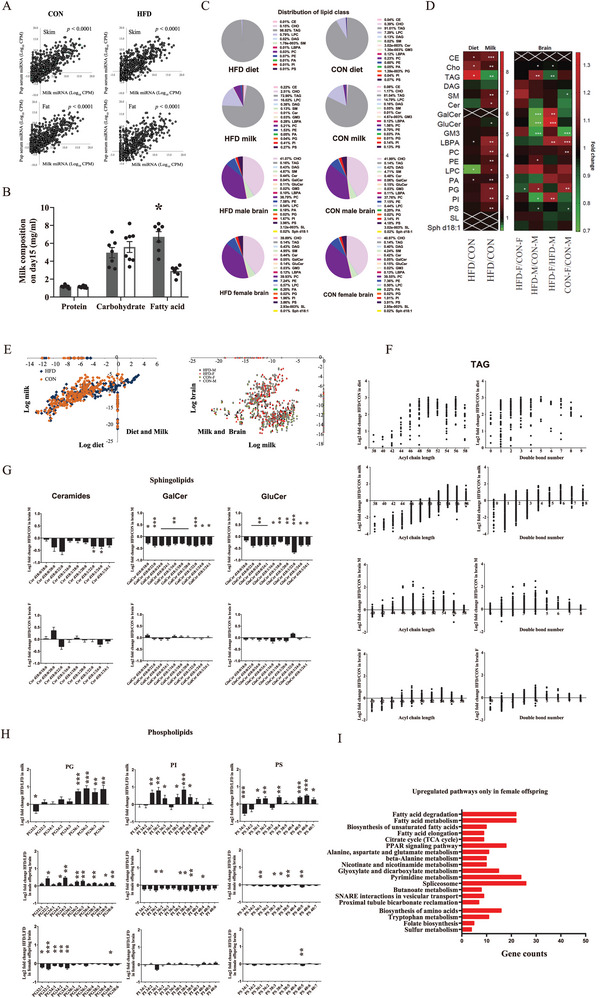
miRNAs and lipidomics profiling in response to maternal HFD feeding during lactation A) miRNAs in milk skim and fat compartments as well as in pup serum (*n =* 8 for milk skim and fat compartments; *n =* 24 for pup serum). B) Macronutrient compositions of milk from mothers fed with an HFD or a CON (*n =* 7–8). C) Percentage distribution of lipid classes in diet (*n =* 2), milk (*n =* 8), male and female pup hypothalami (*n =* 7) on postnatal day 15. D) Detailed lipidomics analysis of diet (*n =* 2), milk (*n =* 8), and male and female pup hypothalami (*n =* 7). E) The correlation between lipid species in diet and in milk (left) as well as lipid species in milk (*n =* 8) and in pup hypothalami (right) (*n =* 7). F) TAG fold change (HFD versus CON) in diet (*n =* 2), milk (*n =* 8), and male and female pup hypothalami (*n =* 7). G,H) Sphingolipids fold change (HFD versus CON) in milk (*n =* 8), and male and female pup hypothalami (*n =* 7). I) KEGG pathway analysis showed upregulated pathways that are only enriched in female pup hypothalami (*n =* 5). Values are represented as mean ± SEM and analyzed by unpaired Student's t‐test. Differences with *p <* 0.05 were considered significant. *p < 0.05, **p < 0.01.

The developmental programing effects observed in offspring pups were not caused by maternal HFD directly. While female attentiveness to her pups has been shown to strongly influence their development, we have already shown that female attentiveness in mice is not affected by HFD feeding in the mother.^[^ ^49^ ^]^ Hence, the dominant interface between the mother and her offspring when exposed to HFD was via the milk. Two critical questions need to be addressed to understand the effects of milk on the offspring's brain development. First, how does the maternal dietary fat intake affect the milk composition? Second, what components in milk might affect hypothalamic development? Milk miRNAs have been proposed to play important roles in regulating offspring development.^[^ ^50^ ^]^ We analyzed the miRNAs in both fat and skim fraction of milk, as well as in pup serum. Across the 447 different miRNAs found, there was a very strong correlation between their abundance in fat and skim fractions (Table S1 and Figure S3A, Supporting Information). We then performed a correlation analysis between miRNAs in milk and in pup serum to determine the possibility of miRNA transfer from milk to pups. The results showed significantly positive associations (**Figure** **6**A), suggesting a possibility of direct transfer of miRNA. 23 miRNAs in milk (Figure S3B and Table S2, Supporting Information) and 51 miRNAs (Table S2, Supporting Information) in pup serum were found to be significantly affected by maternal HFD (*p <* 0.05), with most elevated by maternal HFD. The gene pathways targeted by these miRNAs were analyzed and indicated these miRNAs could downregulate 5‐HT receptor pathway components (Figure S3C,D, Supporting Information), which we observed previously to be affected by maternal high‐fat feeding (Figure 2). These associations suggested that miRNAs in milk might play some role in affecting brain development and brain gene expression.

Only fatty acid (*p =* 0.016) but not protein (*p =* 0.777) or carbohydrate (*p =* 0.580) levels in milk were significantly increased by maternal HFD (Figure 6B). By the detailed lipidomics, the percentage distributions of lipid species were determined (Figure 6C) and multiple comparisons in milk and brain related to diet or sex are presented (Figure 6D,E). As expected, HFD contained more triacylglycerides (TAG) (*p =* 0.002), cholesterol (CHO) (*p =* 0.005), and ceramide (CE) (*p <* 0.05); and only lysophosphatidylcholine (LysoPC) was lower in HFD compared to the CON diet (*p <* 0.05) (Figure 6D and S4A). The major component of milk was TAG (81.04% for CON and 72.9% for HFD, Figure 6C). Maternal HFD resulted in substantial changes in nearly all the detectable lipid species in milk, including unexpectedly lower levels of TAG (*p =* 0.006), and elevated levels of CE (*p <* 0.0001), CHO (*p <* 0.05), and most classes of polar lipids (phospholipids (PGs) and sphingolipids (PSs), *p <* 0.05) (Figure 6D,F,H and Figure S4B, Supporting Information). In contrast to the substantial changes observed in milk, less significant changes across the components were observed in pups’ hypothalami in response to maternal HFD (Figure 6D). A sexually dimorphic hypothalamic response was observed with HFD males having higher unsaturated TAG in the hypothalamus and significant reductions in sphingolipids when their mothers were fed HFD, while the female offspring hypothalamic lipid profiles for these species remained unaffected by maternal HFD (Figure 6F,G). Hypothalamic PGs in males were significantly elevated by maternal HFD, while they were significantly reduced in females (Figure 6H). Hypothalamic phosphoinositides (PIs) and PSs in males were reduced by maternal HFD but unchanged in females (Figure 6H). A KEGG pathway enrichment analysis for differentially expressed genes (DEGs) found in offspring hypothalamus revealed male and female offspring shared 21 upregulated pathways (Figure S4C, Supporting Information) and 75 downregulated pathways (Figure S4D, Supporting Information). There were 4 pathways exclusively upregulated in male offspring (Figure S4E, Supporting Information) and 5 pathways exclusively downregulated in male offspring (Figure S4F, Supporting Information); while there are 19 pathways exclusively upregulated in female offspring (Figure 6I) and 23 pathways exclusively downregulated in female offspring (Figure S4G, Supporting Information). Interestingly, for the exclusively upregulated pathways in females, most were related to metabolism. Of these fatty acid metabolism‐related pathways including fatty acid degradation, fatty acid metabolism, biosynthesis of unsaturated fatty acids, citrate cycle, and PPAR signaling pathway are highly enriched, suggesting an adaptive protection response in fatty acid metabolism in female offspring in response to maternal HFD challenge. This finding was consistent with the RER change in HFD female offspring (Figure S1J, Supporting Information), further confirming the idea that HFD females were better able to elevate their fatty acid metabolism in response to maternal HFD challenge.

## 3 Discussion

In rodents consumption of diets with approximately 50–60% fat, 20% protein, and 20–30% carbohydrates often lead to the development of obesity.^[^ ^51^ ^]^ Similar dietary compositions are also implicated in human weight gain,^[^ ^9^ ^]^ but in both rodents and humans not all individuals eating these diets become obese.^[^ ^21^, ^52^, ^53^ ^]^ In recent years, the contribution of early life developmental programing by maternal over‐consumption of dietary fat to obesity susceptibility in later life has also been revealed by studies using rodent models.^[^ ^16^, ^21^, ^32^, ^45^, ^54^ ^]^ However, a potential sex difference in these impacts remains controversial. For example in mice, some studies showed more obesity susceptibility in male offspring,^[^ ^15^, ^45^, ^55^, ^56^ ^]^ while others showed the opposite result with more obesity susceptibility in females.^[^ ^14^, ^57^ ^]^ This is possibly due to differences between studies including experimental design, diet, mouse strain, and the age and the method by which the offspring susceptibility was assayed. In these studies, some exposed the mother during both prenatal and early postnatal periods,^[^ ^15^, ^55^, ^56^, ^57^ ^]^ but others included manipulation only in lactation.^[^ ^14^, ^45^ ^]^ Moreover, different mouse strains including C57BL/6,^[^ ^55^, ^56^, ^57^ ^]^ CD1,^[^ ^14^, ^15^ ^]^ swiss mice^[^ ^45^ ^]^ were used.

Here, using C57BL/6 mice, we characterized the effect of maternal overconsumption of dietary fat exclusively during lactation on susceptibility to development of diet‐induced obesity in both male and female offspring mice during adulthood. Maternal HFD during lactation increased adiposity of early postnatal offspring mice independent of sex. More importantly, our results showed that maternal HFD during lactation increased obesity risk later in life in males, but not females. These results are consistent with the findings using Swiss mice and also with manipulation of the diet only during lactation.^[^ ^45^ ^]^ Of note, this latter study indicated that the maternal diets between 41.7% and 66.6% fat during lactation all yielded similar effects on the offspring.^[^ ^45^ ^]^ In addition, some other studies using the C57BL/6 mouse model also revealed that maternal HFD during lactation increased obesity susceptibility in male offspring,^[^ ^54^, ^58^ ^]^ though female offspring were not included in the analysis. Maternal HFD during both pregnancy and lactation in C57BL/6 mice also leads to greater obesity susceptibility in male offspring,^[^ ^56^ ^]^ together with studies that are performed with maternal HFD manipulation only during lactation, suggesting that lactation might be more critical than pregnancy for maternal programing. It has been revealed that a similar sex‐differential effect programed by maternal HFD during lactation also occurs in rats.^[^ ^13^ ^]^ Our data revealed a sex‐differential effect with male offspring being more susceptible to obesity in response to maternal HFD during lactation than females, which probably reflects a common phenomenon in rodents.

The hypothalamus plays a major role in body weight regulation.^[^ ^23^, ^24^ ^]^ The molecular signatures in the hypothalamus in response to maternal HFD were investigated by RNA‐seq in both male and female offspring mice at postnatal day 15. Day 15 is a representative day of peak lactation period, at which the mothers have a great energy demand to sustain the accelerated development of the offspring mice, and thus represented as a very critical and sensitive period to environmental perturbations.^[^ ^59^ ^]^ Interestingly, the pathway analysis output showed that 5‐hydroxytryptamine (5‐HT or serotonin) receptor pathway was downregulated by maternal HFD in both males and females, but more so in females in several 5‐HT receptors including HTR1D, HTR4, HTR5A, and HTR6. It is unclear whether these receptors in the hypothalamus are related to body weight regulation. However, studies have shown that the hypothalamic 5‐HT system plays an anorectic role at least through its receptors including HTR2C, HTR1A, and HTR1B.^[^ ^60^, ^61^, ^62^, ^63^ ^]^ For example, HTR2C deficiency specifically in POMC neurons promoted hyperphagia and HFD‐induced obesity.^[^ ^60^ ^]^ Thus, the downregulation of these 5‐HT receptors may act as a common cause of the increased risk of obesity in offspring mice independent of sex. One study in rats revealed a similar 5‐HT receptor (HTR2C and HTR1A)‐mediated mechanism underlying the hypothalamic programming of obesity susceptibility by maternal protein restriction during gestation.^[^ ^64^ ^]^ Body weight is affected by both hedonic and homeostatic hunger systems.^[^ ^65^ ^]^ Hypothalamic dopamine and opioid‐related circuitry were implicated in affecting animals’ preference for palatable foods through reward mechanisms.^[^ ^66^, ^67^ ^]^ We found that the expression of genes related to opioid and dopamine signaling was downregulated by maternal HFD in females but not in males. In addition, NPY (a primary hunger‐promoting gene) and its receptors showed much more downregulation in females compared with males in response to maternal HFD during lactation. Although the expression of POMC (a primary hunger‐suppressing gene) was more elevated in males by maternal HFD, their downstream‐related receptor MC4R did not show a significant difference (Figure 2B). Thus, female offspring mice were much more protected from obesity development at least though the inhibition of some reward and hunger‐promoting genes, suggesting a central molecular mechanism underlying the sex differential effect in obesity susceptibility by maternal programing.

NPY/AgRP and POMC/CARTPT neuron populations in the ARC as well as their balance are mainly established in utero in mice.^[^ ^41^ ^]^ Postnatal hypothalamic neurogenesis and neurotrophic development have been found to contribute to their turnover and balance.^[^ ^42^ ^]^ However, to date, little is known about how the early life over‐nutrition environment contributes to this balance. In the present study, we showed that maternal HFD during lactation increased the ratio between NPY and POMC neurons in both male and female offspring by affecting the turnover of the neurons formed at the embryo stage and the early postnatal stage, but overall, to a more extent in males. These changes likely contribute to the greater obesity risk in male adult offspring when they encounter an HFD.

Mother and offspring interact in complex ways but overall the main factors affecting offspring development are probably maternal attentiveness^[^ ^68^ ^]^ and the milk composition. A recent study has shown that maternal HFD during lactation doesn't affect maternal attentiveness in mice.^[^ ^49^ ^]^ Thus, the neurodevelopmental effects observed in offspring mice are more likely to stem from altered milk composition. Lipids and miRNAs in milk have been found to affect the development of offspring.^[^ ^50^, ^69^ ^]^ Milk can provide all the dietary essential fatty acids to support the development of offspring as signaling molecules or sources of energy, and some of them are critical to normal brain/neuron development.^[^ ^69^, ^70^ ^]^ Milk lipids can be directly affected by maternal diet during lactation in mice.^[^ ^71^ ^]^ We showed only the lipid content of milk was affected (upregulated) by maternal HFD, but not other macronutrient components (protein and carbohydrate). Previous studies have suggested lipid content of milk may be affected not only by the diet but also by maternal obesity.^[^ ^72^ ^]^ However, as reported in a previous study,^[^ ^49^ ^]^ our dietary manipulation, during only lactation, did not lead to obesity development in mothers, and no significant difference in maternal body weight was observed at weaning. Therefore, milk lipids are likely to more reflect the diet than maternal obesity status. One interesting question is whether maternal dietary lipid compositions are directly transferred to milk. In fact, we found that most lipid species in milk were affected by maternal HFD during lactation not necessarily by a direct transfer but also through impacting lipid metabolism. Some lipid species existed in the diet but were excluded from milk, while some found in the milk were absent from the diet (Figure 6E). Of note, TAG levels were found to be reduced in the milk of mothers fed HFD. Lipids are major components of the brain, and their metabolism plays critical roles in brain development related to the regulation of neural stem cells/neurogenesis.^[^ ^73^, ^74^ ^]^ The role of hypothalamic sensing of fatty acids in mediating energy balance has been previously revealed.^[^ ^75^ ^]^ Males and females showed a sex‐differential change in compositions of sphingolipids and phospholipids in response to maternal HFD. Sphingolipids including ceramides, GalCer, and GluCer were downregulated in the hypothalamus in males but not in females. Studies have shown that these sphingolipids are crucial for normal brain/neuronal function. For example, one study showed that downregulation of GluCers in the brain by GluCer synthase deletion leads to neural defects in mice after birth.^[^ ^76^ ^]^ Meanwhile, GluCer synthase inhibitor was found to have inhibitory effects on neurite growth in PC12 cells.^[^ ^77^ ^]^ Another study showed that GalCer supplementation could improve neuropathological parameters impaired in CLN3 disease with impaired GalCer transport in brain.^[^ ^78^ ^]^ Phospholipids are important components forming the membrane lipid bilayers of neurons, thus providing structural integrity for intracellular and cell surface membrane proteins.^[^ ^79^ ^]^ Males showed more changes in phospholipids including PG, PI, and PS. Dysregulated lipid homeostasis in phospholipids is associated with the development of neurological disorders.^[^ ^79^ ^]^ Overall, lipidomic profiling from both mothers and pups revealed that elevated TAG in the high‐fat diet cannot be responsible for the negative effects observed in brain development caused by maternal HFD, as TAG levels were actually reduced in the milk of mothers fed HFD. The changes in sphingolipids and phospholipids in response to HFD feeding of the mother were very different between male and female offspring and may mediate some of the impact of the milk on their brain development. Emerging evidence has shown that milk miRNAs can impose metabolic and immunologic effects on developing offspring.^[^ ^50^, ^80^ ^]^ A human study has shown that several abundant miRNAs in milk were affected by maternal diet.^[^ ^80^ ^]^ miRNAs are stable in milk as they can be packaged within protective vesicles.^[^ ^81^ ^]^ In this form, they might be able to be transferred to the offspring by digestive absorption.^[^ ^82^ ^]^ Our results showed that miRNAs in milk were positively correlated with miRNAs in offspring serum, also suggesting the possibility of miRNAs transfer (Figure 6A). Maternal HFD elevated several miRNAs in milk, which were predicted to downregulate the gene expression of several 5‐HT receptors (Figure 6C), similar to findings from the miRNA profile of offspring serum (Figure 6D). Thus, part of the hypothalamic gene expression response by maternal HFD might be affected via miRNAs.

Hypothalamic development during the perinatal period can be easily affected by neuroendocrine‐related hormones (e.g., leptin, insulin, and ghrelin) and nutritional changes (e.g., maternal obesity and HFD).^[^ ^25^, ^27^, ^31^, ^33^, ^34^, ^35^, ^37^, ^83^ ^]^ For example, the adipocyte‐derived hormone leptin has been found to play a neurotrophic role in the development of the hypothalamus during the neonatal period,^[^ ^35^ ^]^ while neonatal ghrelin (a stomach‐derived hormone) exposure blocked the neurotrophic effect of leptin. Hence together these hormones may program the development of hypothalamic feeding circuits.^[^ ^37^ ^]^ Interestingly, the hypothalamic response to leptin and ghrelin can be impaired by maternal/neonatal overnutrition.^[^ ^83^ ^]^ These metabolic hormones in early postnatal offspring can be affected by their nutritional status and accelerated body weight gain, thus may play important roles in the (sex differential) hypothalamic developmental programing.^[^ ^84^, ^85^ ^]^ The sex differences in these kinds of developmental programing of metabolism related to hypothalamic control have also been observed.^[^ ^84^ ^]^

In summary, we found that maternal HFD exposure during lactation increased susceptibility to the development of obesity in adult male offspring mice, but not in females. These findings support the suggestion that early life programming changes due to maternal over‐nutrition may contribute to obesity development in adulthood. The molecular, cellular/neuron population changes in the hypothalamus in early life set a more obese tone in male offspring mice compared to females. Concomitantly, we also revealed sex differences in hypothalamic lipid metabolism in the early postnatal hypothalamus in response to maternal HFD exposure. Overall, our work suggests the existence of sexual dimorphism with regard to how a maternal HFD during lactation affects neurodevelopment in hypothalamic structure and function in the offspring mice. Tracking these early life changes centrally provides insight into understanding the cause of obesity from its roots and into strategies in maternal dietary intervention during critical period for offspring development, thus preventing the susceptibility to obesity development in adult life.

### 3.1 Limitations of Study

Substantial developmental differences in brain exist between mice and humans. For example, neural circuits in the hypothalamus are fully developed during pregnancy in humans, while they continue to develop during lactation in mice. In addition, human pregnancy represents a much longer period, during which other factors in addition to diet might affect offspring development, such as dramatic weight gain. Thus, we should be cautious when extrapolating these findings to humans. Although our study clearly showed molecular and cellular changes in hypothalamus in response to maternal HFD that may mediate the susceptibility to obesity development in adult life, it cannot be excluded that other brain regions may also contribute to this maternal programming. In addition, studies have shown that the early life programing of other metabolic organs such as brown adipose tissue (BAT) by maternal dietary manipulation may also contribute to later life obesity development.^[^ ^86^, ^87^ ^]^ However, whether these early life remodeling events in response to maternal HFD during lactation also exist in a gender‐specific way may warrant further study.

## 4 Experimental Section

### Ethical Statement

All animal experiments were performed following an internal ethical review in accordance with the guidelines of the Institute of Genetics and Developmental Biology and Shenzhen Institutes of Advanced Technology, Chinese Academy of Sciences. The approval numbers for the experiments were AP2016018 and SIAT‐IACUC‐200923‐YYS‐JRS‐A1455.

### Mice

All the C57BL/6 mice (aged 8 weeks old) used for mating in the present study were purchased from the Beijing Vital River Laboratory animal center and housed in the animal facility of the Institute of Genetics and Developmental Biology (IGDB), Chinese Academy of Sciences. They were maintained in SPF facility conditions with a 12‐hr light/dark cycle (lights on at 7:30 a.m. and off at 7:30 p.m.) at an ambient temperature of 23 ± 1 °C with access to a standard control diet (D12450B, Research Diets; for calories, 3.85 kcal/g, 10% from fat, 20% from protein, 70% from carbohydrate) and water ad libitum. All mice were singly housed and acclimated to the environment for 2 weeks before starting the experimental procedures.

### Experimental Design

*EXPERIMENT 1: To explore how the maternal HFD feeding during lactation affects neonatal pups’ hypothalamic neurogenesis*

Thirty female C57BL/6 mice aged 8 weeks old were obtained and fed a standard control diet. Two weeks after the acclimation, they were bred with male C57BL/6 mice fed a standard control diet, and the male mice were removed two days later. All pregnant female mice were maintained on the standard control diet throughout pregnancy. Maternal body weight was monitored every 3 days during pregnancy.

After parturition (defined as day 0 of lactation), the lactating mice with 6 to 9 pups were selected and randomly divided into two groups: one group was continuously maintained on the standard control diet (defined as CON group; *N =* 8); the other group was switched to a standard high‐fat diet (defined as HFD group; *N =* 7) (D12451, Research Diets; for calories, 4.73 kcal/g, 45% from fat, 20% from protein, 35% from carbohydrate). During lactation, maternal body weight, food intake, and litter mass were measured daily. Food intake was calculated by subtracting the food in the food hopper on the second day from the food placed in the food hopper on the first day. One male pup and one female pup were randomly chosen from each litter on lactation day 9, day 15, and day 21, respectively. They received three intraperitoneal injections of BrdU (100 mg k^−1^g body weight) 3 hours apart during the light cycle and the last injection was at 15:00. Three hours later (i.e., at 18:00), these mice were anesthetized by administration of sodium pentobarbital (30 mg k^−1^g body weight) and perfused transcardially with 0.9% NaCl followed by 4% paraformaldehyde solution (PFA). The brains were dissected and processed for later analysis (i.e., post‐fixed in 4% PFA at 4 °C overnight, and then transferred to 30% sucrose for 48 h; all brains were then embedded in OCT compound (SAKURA, Tissue‐Tek O.C.T. Compound, Order Number 4583), and stored at −80 °C). Meanwhile, the subcutaneous and visceral gonadal fat were dissected and weighed for mice on day 15 and day 21.

*EXPERIMENT 2: To explore how the maternal HFD during lactation affects the HFD‐induced obesity development in both male and female adult offspring mice*

The remaining pups (for HFD group, 9 males and 9 females; for CON group, 8 males and 9 females) in Experiment 1 were used for this experiment. After weaning, they were maintained on the standard control diet. At the age of 12 weeks, these mice were switched to HFD for 11 weeks to induce obesity, thus forming two groups both in males (defined as group HFD‐M and CON‐M) and females (defined as group HFD‐F and CON‐F). Body weight and food intake were measured daily. The body fat mass was monitored weekly by EcoMRI Body Composition Analyzer. The oxygen (O_2_) consumption (mL mi^−1^n), carbon dioxide (CO_2_) production (mL mi^−1^n), respiratory exchange ratio (RER = VCO_2_/VO_2_), and physical activity (Counts) were measured by TSE PhenoMaster system after 10 weeks of HFD exposure.

*EXPERIMENT 3: To explore how the maternal HFD during lactation affects the fate of the newborn cells in pup hypothalamus*

Thirty female C57BL/6 mice aged 8 weeks old were used in this experiment, and the breeding procedure was the same as in EXPERIMENT 1. After parturition (defined as lactation day 0), lactating mice with 6 to 9 pups were also selected and randomly divided into two groups: one group was maintained on the standard control diet (CON group, *N =* 8); and the other group was switched to the standard HFD (HFD group, *N =* 7). One male pup and one female pup were randomly chosen on lactation day 9, day 15, and day 21, and received 3 intraperitoneal injections of BrdU same as in Experiment 1. Twenty‐one days after BrdU injection, these mice were sacrificed, and brains were collected and processed as in Experiment 1. The diagram for experimental design is shown in Figure 5AM.

*EXPERIMENT 4: To explore how the maternal HFD during lactation affects the neuron fate of the embryo‐born cells in pup hypothalamus*

As in Experiment 1 and 3, another thirty female C57BL/6 mice aged at 8 weeks old were obtained and fed the standard control diet. Two weeks after acclimation, the female mice were then bred with normal male C57BL/6 mice fed the standard control diet. Noon of the same day that vaginal plug discovery was assumed to be embryonic day 0.5 (E0.5), and then the male mice were removed. All pregnant female mice were maintained on the standard control diet throughout pregnancy. On E12.5, the pregnant mice received two injections of BrdU intraperitoneally at 3 hours apart (100 mg k^−1^g body weight, sigma).

After parturition (defined as lactation day 0), lactating mice with 6 to 9 pups were selected and randomly divided into two groups: one group was maintained on the standard control diet (CON group, *N =* 7); the other group was switched to the standard HFD (HFD group, *N =* 7). On lactation day 9, day 15, and day 21, one male pup and one female pup were randomly chosen and sacrificed; the brains were dissected and processed as in Experiments 1 and 3 for analysis. The diagram of the experimental design is shown in Figure 3Z.

### Immunofluorescence Staining

A serial 14 µm‐thick frozen sections spanning the hypothalamus were prepared using a cryostat (10 series; Leica CM1950, Leica Microsystems). Slides with brain sections were stored at −80 °C until further use. For BrdU immunostaining, sections were incubated in 2N HCl at 37 °C for 1 h, washed with 0.01 m phosphate‐buffered saline (PBS; P4417, Sigma), and then incubated in primary rat anti‐BrdU antibody (1:200, ab6326, Abcam) diluted in 1% BSA with PBS plus 0.1% Triton X‐100 (blocking buffer) overnight (12 to 16 hours) at 4°C. Sections were washed with PBS plus 0.1% Triton X‐100, followed by a reaction with secondary antibody of anti‐Rat IgG H&L Alexa Fluor 647 (1:400, ab150159, Abcam) in blocking buffer at room temperature for 2 hrs, washed with PBS, and 4′,6‐diamidino‐2‐phenylindole (DAPI) was added to visualize the cell nuclei. Stained sections were examined using LSM710 confocal microscopy (Zeiss). Nuclear localization of BrdU^+^ cells was verified by colocalization with DAPI. Regions of the hypothalamus, including the median eminence (ME), arcuate nucleus (ARC), ventromedial nucleus (VMH), and dorsomedial nucleus (DMH) were defined according to the mouse brain atlas of Paxinos and Franklin (2019).^[^ ^88^ ^]^ The number of BrdU^+^ cells in 3 consecutive central hypothalamic sections containing the ME, ARC, VMH, and DMH regions were counted manually, and then the number of BrdU^+^ cells was averaged across sections to provide a single data point for each mouse.

For BrdU and NeuN/NPY/POMC/GFAP/Iba1 double immunostaining, sections were incubated in 2N HCl at 37°C for 1 h, washed in PBS, and then incubated with primary rat BrdU antibody (1:200, ab6326, Abcam) and primary mouse monoclonal NeuN antibody (1:1000, ab104224, Abcam), or primary rabbit Neuropeptide Y (NPY) antibody (1:1000, ab10980, Abcam), or primary rabbit pro‐opiomelanocortin (POMC) antibody (1:1000, ab254257, Abcam), or primary rabbit glial fibrillary acidic protein (GFAP) antibody (1:500, ab7260, Abcam), or primary rabbit ionized calcium‐binding adaptor molecule 1 (Iba1) antibody (1:500, ab153696; Abcam) diluted in 1% BSA with PBS plus 0.1% Triton X‐100 (blocking solution) overnight at 4 °C, respectively. Sections were then washed with PBS plus 0.1% Triton X‐100, followed by reaction with corresponding secondary antibodies goat anti‐rat IgG H&L Alexa Fluor 647 (1:400, ab150159, Abcam) and donkey anti‐mouse IgG H&L Alexa Fluor 488 (1:400, ab150105, Abcam) or goat anti‐rabbit IgG H&L Alexa Fluor 488 (1:400, ab150077, Abcam) in blocking buffer at room temperature for 2 hours, washed with PBS to visualize. Three consecutive sections in each slide were counted for BrdU^+^, NeuN^+^, NPY^+^, POMC^+^, GFAP^+,^ and Iba1^+^ cells, and the number of cells in these sections was averaged as a single data point for each mouse.

### Milk Collection and Composition Measurements

On day 15 of lactation, the dams from Experiment 1 were separated from the pups for 2 hours to collect milk. They received 2 IU/kg of oxytocin (O3251, Sigma) intraperitoneally. Oxytocin can act on the mammary glands of lactating females to stimulate the release of milk. After the injection of oxytocin, the dams were anesthetized with an isoflurane machine suited for mice. The milk collection was performed by two researchers: one researcher to hold the anesthetized mouse when manually expressing the milk, and another researcher to collect the milk.^[^ ^89^ ^]^ The collected milk (ranges ≈ 50 to 100 µL) was stored at −80 °C until assay. After the dams were fully recovered from the anesthesia, the pups were put back into the same cage.

Total protein, carbohydrate, and free fatty acid in the milk were determined using the commercial kits according to their instructions (Pierce BCA Protein Assay Kit (23225) from Thermo Scientific, Total Carbohydrate Assay Kit (MAK104), and free fatty acid kit (MAK044) from Sigma).

### Targeted Lipidomics

Two diet samples from both the standard control diet and HFD diet, 8 milk samples from CON group and 7 milk samples from HFD group, and 12 hypothalamic section samples (6 males and 6 females) from CON group and 14 hypothalamic section samples (7 males and 7 females) from HFD group were used for lipidomics analysis. The experimental procedures for lipiodmics analysis were described in a previous study.^[^ ^90^, ^91^ ^]^

### RNA‐seq Transcriptome Analyses

Using the same procedures as in Experiment 1, 10 pregnant female mice were prepared and fed on the standard control diet. Similarly, after parturition, they were divided into two groups, 5 mice were maintained on the standard control diet (CON group), and another 5 mice were switched to the standard HFD (HFD group). On day 15 of lactation, 5 male pups and 5 female pups from both CON group and HFD group were euthanized by an overdose of CO_2_. Immediately, the brains were dissected and stored at −80 °C for hypothalamic RNA extraction.

Total RNA was extracted from the hypothalamus of these stored brains using an RNeasy lipid tissue mini kit (Qiagen, 74804) according to the manufacturer's protocol. The quality of RNA samples was determined by the Agilent 2100 Bioanalyzer (RIN Score 8 or higher). RNA sequencing was performed using the Illumina NextSeq 500 sequencer as described in the previous study.^[^ ^51^ ^]^ FastQC (http://www.bioinformatics.babraham.ac.uk/projects/fastqc/), TopHat‐HTSeq pipeline, and edgeR (version 3.12.0, R version 3.2.2) were used for confirming the sequencing quality, reads mapping and obtaining the count data, and differentially gene expression analysis. Ingenuity Pathway Analysis (IPA, Ingenuity Systems; http://www.ingenuity.com/) was used to determine the significantly affected pathways. KEGG pathway enrichment analysis for upregulated and downregulated DEGs was performed using DAVID bioinformatics resources.

### miRNA‐seq of Milk and Pup Serum

Experiment was performed on 24 lactating females that were fed either HFD or CON diets (*n =* 12 mice per group), following the breeding and feeding protocols detailed above. Milk samples and pup sera were collected on day 15 of lactation. To obtain milk samples, females were separated from pups for 2 h, and injected with 2 IU/kg of oxytocin and milk manually as described above. Milk samples were immediately frozen and stored at −80 °C until further processing. Following milk collection, all pups within each litter were decapitated to collect trunk blood samples, which were then pooled to have one blood sample per litter. Blood samples were clotted and centrifuged to obtain sera, which were immediately frozen and stored at −80 °C until RNA extraction.

The milk was thawed on ice and samples from three females on the same diet were pooled to have ≈1 mL of milk for analysis. As a result, each diet manipulation was represented by 4 replicate pools of milk, generating 8 milk pools in total with milk from 24 females (2 diets × 4 pools × 3 females per pool). The milk pools were then centrifuged at 17 000 × g for 30 min at 4 °C to separate milk lipid from skim milk. Total RNA including small RNAs (such as miRNAs) was isolated from 8 milk lipids and 8 skim milk samples using a commercially available RNA isolation kit (mirVana miRNA Isolation Kit, Ambion), following the manufacturer's instructions.

The pup serum (24 samples representing 24 litters) was thawed on ice and ≈200 mL of each serum was used for RNA isolation. Cell‐free total RNA (including miRNAs) from serum samples was extracted using miRNeasy Serum/Plasma Kit (QIAGEN), according to the manufacturer's instructions.

Sample QC, library preparation, and sequencing were performed at Glasgow Polyomics (University of Glasgow, UK). Total RNA in 40 samples was quantified by spectrophotometry (NanoDrop Technologies, Wilmington, DE, USA), with 39 samples confirmed to have sufficient RNA to proceed with library preparation (15.4–218.8 ng µL^−1^), while 1 sample (milk lipid from the HFD group) was removed due to a low concentration of total RNA (4.7 ng µL^−1^). The integrity and concentration of small RNA (890.4–38938.1 pg µL^−1^) and miRNA (646.8–20124.5 pg µL^−1^) in 39 samples were confirmed by electrophoresis using the Bioanalyzer Small RNA assay (Agilent Technologies, Santa Clara, CA, USA).

The libraries were constructed using the Illumina TruSeq Small RNA Sample Preparation Kit (Illumina, San Diego, CA, USA), according to the manufacturer's instructions. The 50‐bp single‐end sequencing was performed on the HiSeq 500 Sequencing System (Illumina, San Diego, CA) at a sequencing depth of ∼50 million reads per library. The raw reads in BCL format were converted to FastQ format with bcl2fastq2 Conversion Software v2.19.1 (Illumina, San Diego, CA).

To assess the quality of the sequencing data, reads were analyzed with FastQC v0.11.8 and trimmed with Cutadapt v1.10. Filtered reads were then mapped to the mouse genome version GRCm38 using bowtie version 1.0.0. Reads aligned to miRNA regions (defined by the miRbase annotation file) were counted using bedtools v2.23.0. Only counts for mature miRNAs were used for further analysis.

Analysis of differential expression of mature miRNAs was performed using DESeq v1.32.0. Raw counts per million (CPM) for 2045 miRNAs were normalized using a trimmed mean of M‐values (TMM). The normalized CPM values were then used for correlation analysis and differential expression analysis, with the contrasts set up to compare milk fat, skim milk, and pup sera in the mothers on HFD versus CON diets. Specifically, the contrasts were based on 3 milk lipid HFD versus 4 milk lipid CON samples, 4 skim milk HFD versus 4 skim milk CON samples, and 12 pup serum HFD versus 12 pup serum CON samples. Differentially expressed miRNAs were identified at *p*‐value **<** 0.05 and absolute *log*_2_ FC > 0.5. The genes predicted to be targeted by the differential milk and pup serum miRNAs were mapped to the hunger signaling gene network.

### Statistical Analysis

Data were analyzed using the R platform, IBM SPSS 17.0, GraphPad Prism 7.0, and Microsoft Excel. Differences in adult male and female offspring body weight, body fat, energy intake, oxygen consumption RER, physical activity, maternal body mass, energy intake, and average litter mass between CON and HFD groups were analyzed by repeated‐measures ANOVA. Body fat mass of pups (subcutaneous, visceral, and total fat mass) on day 15 and day 21 between the two groups was analyzed by independent unpaired Student's t‐test. Other parameters between treatment groups including BrdU^+^ cells, NeuN^+^, NPY^+^, POMC^+^, GFAP^+,^ or Iba1^+^ neurons and those neurons co‐labeled with BrdU, and the ratios between NPY^+^BrdU^+^ neurons to POMC^+^BrdU^+^ neurons, and compositions of milk were analyzed by independent unpaired Student's t‐test. Lipid species in diet, milk, and brain were also analyzed by independent unpaired Student's t‐test. All values were expressed as the mean ± SEM. Differences between groups were considered statistically significant at *p <* 0.05.

## Conflict of Interest

The authors declare no conflict of interest.

## Author Contributions

Y.X., D.Y., L.W.and E.K. contributed equally to this work. J.R.S., Y.X., and D.Y. were responsible for conceptualization. Methodology and investigation were carried out by Y.X., D.Y., L.W., E.K., M.M., L.L., Y.H., C.N., X.L., A.D., L.S., and G.S. J.R.S. provided supervision. Y.X. and D.Y. were responsible for the original draft, while J.R.S., Y.X., and D.Y. participated in the review and editing process.

## Supporting information

Supporting Information

## Data Availability

The data that support the findings of this study are available from the corresponding author upon reasonable request.
