# Supplementary material for: Maternal High Fat Diet in Lactation Impacts Hypothalamic Neurogenesis and Neurotrophic Development, Leading to Later Life Susceptibility to Obesity in Male but Not Female Mice
Source: Adv Sci (Weinh). 2023 Oct 22;10(35):2305472. doi: 10.1002/advs.202305472 (PMC10724448; doi:10.1002/advs.202305472)
Supplement: Supplementary file 1 — Supporting Information [file ADVS-10-2305472-s001.pdf]

ADVANCED SCIENCE
Open Access

Supporting Information

for *Adv. Sci.*, DOI 10.1002/advs.202305472

Maternal High Fat Diet in Lactation Impacts Hypothalamic Neurogenesis and Neurotrophic Development, Leading to Later Life Susceptibility to Obesity in Male but Not Female Mice

*Yanchao Xu, Dengbao Yang, Lu Wang, Elżbieta Król, Mohsen Mazidi, Li Li, Yi Huang, Chaoqun Niu, Xue Liu, Sin Man Lam, Guanghou Shui, Alex Douglas and John R. Speakman**

Supplementary Table 1 Lists of the miRNAs in both fat and skim fraction of milk, as well as in pup's serum in control diet fed group

miRNA name	Milk skim fraction (CPM: means ± SEM)	Milk fat fraction (CPM: means ± SEM)
MIMAT0000154_mmu-miR-142a-5p	139895.92±7761.41	145165.17±6267.05
MIMAT0028075_mmu-miR-7084-3p	86769.61±1816.96	73079.21±5202.2
MIMAT0000657_mmu-miR-200c-3p	87065.26±1892.67	73323.91±5216.18
MIMAT0000537_mmu-miR-27a-3p	42634.95±2461.6	40747.61±319.75
MIMAT0027977_mmu-miR-7036a-3p	45825.43±1308.65	32057.9±627.08
MIMAT0003473_mmu-miR-133a-5p	38705.42±1520	33711.73±1808.05
MIMAT0028017_mmu-miR-7056-3p	38955.16±693.93	34389.74±1385.32
MIMAT0027753_mmu-miR-6926-3p	23907.24±682.71	33839.75±1104.1
MIMAT0003729_mmu-miR-216b-5p	15843.62±1416.18	17546.83±711.84
MIMAT0027710_mmu-miR-6905-5p	14630.79±1988.92	20895.92±1881.08
MIMAT0027958_mmu-miR-7027-5p	16862.2±642.84	16210.67±2076.9
MIMAT0027737_mmu-miR-6918-3p	11880.41±995.22	7389.54±471.3
MIMAT0000163_mmu-miR-153-3p	12905.23±176.62	14745.95±710.89
MIMAT0028094_mmu-miR-7094-1-5p	9685.43±552.64	9088.78±443.52
MIMAT0000612_mmu-miR-135b-5p	9563±508.85	9135.34±404.19
MIMAT0017324_mmu-miR-669d-3p	7634±247.35	5823±79.96
MIMAT0017169_mmu-miR-215-3p	7662.36±278.5	9897.25±469.2
MIMAT0000558_mmu-miR-325-5p	7266.33±500.84	9971.69±539.27
MIMAT0027998_mmu-miR-7047-5p	6352.16±283.91	8295.24±213.78
MIMAT0022503_mmu-miR-344i	6901.03±1217.48	7771.81±300.11
MIMAT0000140_mmu-miR-128-3p	5727.06±745.99	5668.3±325.92
MIMAT0028088_mmu-miR-7091-5p	5033.93±218.42	4377.78±66.13
MIMAT0028390_mmu-miR-7211-5p	5155.86±476.52	9029.82±658.57
MIMAT0000520_mmu-miR-208a-3p	5914.2±284.46	5238.72±746.06
MIMAT0027941_mmu-miR-7018-3p	5752.99±285.28	5060.25±716.23
MIMAT0004529_mmu-miR-125b-2-3p	4570.16±160.38	3883.21±75.3
MIMAT0000383_mmu-let-7d-5p	4479.15±301.01	4820.99±445.81
MIMAT0025140_mmu-miR-6391	4314.38±117.88	5322.13±194.59
MIMAT0017054_mmu-miR-216a-3p	3751.33±318.56	3963.87±99.8
MIMAT0004668_mmu-miR-138-1-3p	4494.76±122.62	3463.71±114
MIMAT0027734_mmu-miR-6917-5p	3651.93±245.47	6351.32±488.78
MIMAT0022704_mmu-miR-489-5p	2898.26±190.37	3389.09±189.43
MIMAT0001418_mmu-miR-431-5p	2881.23±230.52	3989.68±151.61
MIMAT0027965_mmu-miR-7030-3p	2842.41±203.12	4006.07±140.74
MIMAT0031401_1_mmu-miR-8099	2774.07±208.69	2241.28±97.65
MIMAT0004187_mmu-miR-744-5p	2827.88±120.46	3097.69±206.54
MIMAT0025085_mmu-miR-6342	1953.87±99.08	2195.93±160.05
MIMAT0003485_mmu-miR-455-5p	1889.5±50.98	2606.92±173.38
MIMAT0005858_mmu-miR-1197-3p	1922.25±57.06	1757.91±171.94
MIMAT0027731_mmu-miR-6915-3p	1778.6±126.15	3162.32±167.77
MIMAT0027714_mmu-miR-6907-5p	1483.63±62.06	1139.49±78.55
MIMAT0025113_mmu-miR-6369	2605.65±815.73	843.44±239.2
MIMAT0027712_mmu-miR-6906-5p	2605.65±815.73	843.44±239.2

MIMAT0017256_mmu-miR-700-5p	1354.96±29.28	1336.67±32.83
MIMAT0004573_mmu-miR-293-5p	1859.33±689.33	1185.06±38.56
MIMAT0022360_mmu-miR-5616-3p	1183.93±83	1594.77±49.91
MIMAT0007873_mmu-miR-1896	1295.79±23.25	1612.99±100.24
MIMAT0029814_mmu-miR-7654-5p	1724.86±258.97	1083.68±154.62
MIMAT0005842_mmu-miR-669h-3p	963.8±122.84	1346.58±108.63
MIMAT0014808_mmu-miR-344d-3p	1757.13±548.38	638.33±171.83
MIMAT0028054_mmu-miR-7074-5p	1757.13±548.38	638.33±171.83
MIMAT0014910_mmu-miR-3094-3p	882.59±28.6	1011.99±29.66
MIMAT0025122_mmu-miR-6377	776.87±60.21	1213.36±74.27
MIMAT0025129_mmu-miR-6383	967.26±88.65	1509.88±59.39
MIMAT0019354_mmu-miR-28b	797.92±35.97	835.94±24.85
MIMAT0022377_mmu-miR-5624-5p	990.13±82.65	989.91±108.49
MIMAT0004939_mmu-miR-208b-3p	941.64±62.74	539.61±59.2
MIMAT0014923_mmu-miR-344e-5p	638.54±44.12	604.46±35.82
MIMAT0029800_mmu-miR-7649-5p	781.76±24.76	681.09±40.82
MIMAT0000665_mmu-miR-223-3p	843.49±69.81	435.74±31.62
MIMAT0027946_mmu-miR-7021-5p	760.74±69.78	716.81±42.95
MIMAT0028005_mmu-miR-7050-3p	572.32±111.6	675.81±147.24
MIMAT0031413_mmu-miR-8108	676.72±23.35	733.39±21.64
MIMAT0014864_mmu-miR-3078-5p	625.28±15.15	558.91±10.03
MIMAT0025583_mmu-miR-6538	803.5±81.73	643.96±74.84
MIMAT0014856_1_mmu-miR-3074-5p	758.86±74.93	652.48±73.48
MIMAT0017060_mmu-miR-221-5p	1219.35±317.64	943.33±236.08
MIMAT0004882_1_mmu-miR-466f-3p	497.5±25.34	517.06±18.56
MIMAT0029843_mmu-miR-7668-3p	487±48.28	515.94±26.88
MIMAT0029883_mmu-miR-7681-3p	513.68±37.57	444.16±11.5
MIMAT0027774_mmu-miR-6937-5p	528.06±30.03	537.69±43.03
MIMAT0004758_mmu-miR-463-3p	436.68±30.95	220.46±19.73
MIMAT0028030_mmu-miR-7063-5p	476.93±16.96	599.71±40.7
MIMAT0003467_mmu-miR-688	445.89±29.47	434.45±40.73
MIMAT0000663_mmu-miR-218-5p	374.15±38.39	787.62±65.09
MIMAT0025123_mmu-let-7j	477.78±53.38	363.44±74.31
MIMAT0004580_mmu-miR-34c-3p	452.1±17.85	383.48±9.46
MIMAT0004875_4_mmu-miR-466b-5p	398.16±24.53	442.99±24.17
MIMAT0025138_mmu-miR-378c	413.81±45.13	396.94±10.96
MIMAT0004847_mmu-miR-882	384±11.81	504.68±30.17
MIMAT0017013_mmu-miR-196a-1-3p	369.4±8.88	457.86±11.22
MIMAT0000152_mmu-miR-140-3p	456.62±38.35	248.77±18.42
MIMAT0000216_mmu-miR-187-3p	284.64±14.79	343.51±20.12
MIMAT0000649_mmu-miR-17-5p	535.11±164.31	275.81±78.4
MIMAT0028009_mmu-miR-7052-3p	351.06±14.02	189.46±4.54
MIMAT0031399_mmu-miR-8097	429.36±42.86	442.04±50.57
MIMAT0000529_mmu-miR-20a-5p	237.69±27.45	480.25±36.32
MIMAT0027720_mmu-miR-6910-5p	312.69±65.89	523.99±163.26
MIMAT0000659_mmu-miR-212-3p	270.92±7.7	198.44±14.17

MIMAT0004623_mmu-let-7f-1-3p	312.37±15.48	282.53±25.35
MIMAT0027976_mmu-miR-7036a-5p	313.48±15.87	282.71±25.34
MIMAT0004843_mmu-miR-879-3p	259.53±11.33	355.59±26.91
MIMAT0029815_mmu-miR-7654-3p	418.98±96.78	130.37±20.96
MIMAT0031406_mmu-miR-8102	207.49±16.99	322.81±19.4
MIMAT0005859_mmu-miR-1198-5p	394.56±63.81	76.07±22.98
MIMAT0015646_mmu-miR-3474	225.17±21.6	65.44±3.21
MIMAT0017278_mmu-miR-92b-5p	214.68±13.12	275.89±7.77
MIMAT0022836_mmu-miR-299b-5p	277.29±19.62	176.86±14.99
MIMAT0026642_mmu-miR-3475-5p	204.72±19.81	137.19±9.01
MIMAT0017046_mmu-miR-101b-5p	145.72±15.4	181.94±12.5
MIMAT0027735_mmu-miR-6917-3p	197.95±24.37	201.57±32.97
MIMAT0017018_mmu-miR-16-2-3p	194.14±17.64	289.87±45.2
MIMAT0000706_mmu-miR-362-5p	150.99±22.89	146.09±12.98
MIMAT0000571_mmu-miR-331-3p	127.94±10.95	161.22±4.35
MIMAT0027857_mmu-miR-6977-3p	149.27±5.97	98.19±4.42
MIMAT0017243_9_mmu-miR-669a-3p	144.59±11.85	91.37±8.46
MIMAT0020624_mmu-miR-5116	135.09±20.03	113.91±5.76
MIMAT0000217_mmu-miR-188-5p	113.54±3.96	99.37±2.63
MIMAT0017021_mmu-miR-96-3p	108.49±10.07	112.68±4.37
MIMAT0004940_mmu-miR-511-5p	176.62±26.49	152.69±22.43
MIMAT0028048_mmu-miR-7071-5p	94.24±8.59	121.93±6.34
MIMAT0016995_mmu-miR-182-3p	98.92±6.52	109.12±6.83
MIMAT0025146_mmu-miR-6395	108.45±9.77	55.37±2.97
MIMAT0027798_mmu-miR-6949-5p	105.67±4.52	165.91±12.94
MIMAT0017063_mmu-miR-29b-2-5p	74.18±9.95	74.56±5.93
MIMAT0027921_mmu-miR-7008-3p	120.99±14.74	106.74±8.82
MIMAT0012773_mmu-miR-767	69.13±9.89	77.44±2.39
MIMAT0000517_mmu-miR-192-5p	99.1±11.36	123.61±4.59
MIMAT0016982_mmu-miR-128-1-5p	97.68±4.59	69.17±5.77
MIMAT0027967_mmu-miR-7031-3p	104.76±9.05	66.83±3.43
MIMAT0000536_mmu-miR-29c-3p	73.76±9.67	94.77±3.66
MIMAT0001542_mmu-miR-449a-5p	121.01±17.21	143.64±11.36
MIMAT0004581_mmu-miR-34b-3p	88.14±7.08	89.55±16.47
MIMAT0004661_mmu-miR-28a-3p	85.34±4.54	75.36±6.93
MIMAT0000162_mmu-miR-152-3p	63.75±7.71	94.31±15.64
MIMAT0022356_1_mmu-miR-5615-3p	80.65±9.48	82.11±2.54
MIMAT0007868_mmu-miR-1903	85.41±1.58	67.06±7.76
MIMAT0000378_mmu-miR-300-3p	76.38±4.38	64.33±6.83
MIMAT0025175_mmu-miR-6419	95.69±5.57	78.34±2.84
MIMAT0003471_1_mmu-miR-692	74.03±4.56	62.48±3.64
MIMAT0004635_mmu-miR-92a-2-5p	151.71±57.55	73.86±19.43
MIMAT0000225_mmu-miR-195a-5p	66.79±4.45	87.05±5.54
MIMAT0014876_mmu-miR-3084-5p	66.79±4.45	87.05±5.54
MIMAT0027851_mmu-miR-6974-3p	68.47±3.19	84.68±10.28
MIMAT0004849_mmu-miR-883a-3p	68.47±3.19	84.68±10.28

MIMAT0000647_mmu-miR-107-3p	64.61±5.72	55.72±7.49
MIMAT0004631_mmu-miR-29a-5p	47.28±5.27	46.04±0.88
MIMAT0028047_mmu-miR-7070-3p	73.36±10.83	50.25±5.44
MIMAT0000247_mmu-miR-143-3p	54.36±4.77	92.93±4.4
MIMAT0028133_mmu-miR-7118-5p	67.79±4.31	49.67±4.69
MIMAT0000375_3_mmu-miR-297a-5p	48.85±5	56.54±10.41
MIMAT0027772_mmu-miR-6936-5p	50.68±7.18	43.65±0.82
MIMAT0028074_mmu-miR-7084-5p	30.1±8.87	24.05±7.31
MIMAT0027901_mmu-miR-6999-3p	53.83±2.07	39.24±3.27
MIMAT0027867_mmu-miR-6982-3p	68.1±6.52	73.04±14.12
MIMAT0025126_mmu-miR-6380	35.71±7.68	29.18±3.14
MIMAT0000368_mmu-miR-291a-3p	39.51±4.29	32.04±5.96
MIMAT0027970_mmu-miR-7033-5p	42.83±3.05	47.78±4.51
MIMAT0000664_mmu-miR-219a-5p	38.85±3.93	28.9±2.17
MIMAT0027790_mmu-miR-6945-5p	67.45±7.86	39.11±5.16
MIMAT0027692_mmu-miR-6896-5p	50.04±6.15	24.78±3.42
MIMAT0015643_mmu-miR-3472	37.56±3.58	45.4±3.35
MIMAT0000386_mmu-miR-106b-5p	40.26±3.75	72.2±7.76
MIMAT0017006_mmu-miR-143-5p	35.78±4.13	73.23±5.78
MIMAT0027877_mmu-miR-6987-3p	48.69±4.05	23.96±1.06
MIMAT0027339_mmu-miR-6481	47.61±5.72	21.85±3.56
MIMAT0003783_mmu-miR-615-3p	32.96±3.22	31.23±2.23
MIMAT0003481_mmu-miR-695	48.78±8.03	55.68±2.81
MIMAT0000661_mmu-miR-214-3p	32.54±3.45	41.85±3.96
MIMAT0016984_mmu-miR-132-5p	30.87±5.89	29.84±3.74
MIMAT0028052_mmu-miR-7073-5p	32.86±2.94	34.16±4.13
MIMAT0027934_mmu-miR-7015-5p	92.46±29.21	49.14±15.39
MIMAT0000137_mmu-miR-126a-5p	30.87±5.89	29.84±3.74
MIMAT0000211_mmu-miR-182-5p	39.36±1.9	34.93±2.64
MIMAT0017012_mmu-miR-192-3p	44.34±2.86	141.81±49.13
MIMAT0004870_mmu-miR-453	39.82±2.42	42.59±1.38
MIMAT0017257_mmu-miR-701-3p	42.79±4.77	56.41±2.14
MIMAT0000655_mmu-miR-100-5p	32.01±2.51	41.93±3.01
MIMAT0022985_mmu-miR-5107-3p	38.26±2.31	41.65±1.18
MIMAT0004527_2_mmu-miR-124-5p	49.24±14.51	267.77±144.07
MIMAT0020642_mmu-miR-5131	34.44±2.48	22.32±0.42
MIMAT0028011_mmu-miR-7053-3p	35.72±4	8.93±1.68
MIMAT0028450_mmu-miR-7241-5p	34.44±2.48	22.32±0.42
MIMAT0003409_2_mmu-miR-467a-5p	69.74±13.92	54.77±13.61
MIMAT0000528_mmu-miR-18a-5p	35.33±2.43	24.19±0.73
MIMAT0025115_mmu-miR-6371	37.21±2.41	43.02±4.98
MIMAT0000741_mmu-miR-377-3p	52.19±6.34	57.11±4.98
MIMAT0028013_mmu-miR-7054-3p	39.9±2.98	29.65±3.87
MIMAT0009419_1_mmu-miR-669m-3p	30.39±2.14	45.61±3.03
MIMAT0003711_mmu-miR-652-3p	25.4±2.93	40.74±3.61
MIMAT0014906_mmu-miR-3092-3p	39.16±10.78	31.26±10.02

MIMAT0000381_mmu-miR-34c-5p	33.13±1.81	34.96±3.89
MIMAT0005835_mmu-miR-1b-5p	26.4±2.9	49.57±6.03
MIMAT0000147_mmu-miR-135a-5p	31.73±4.69	43.22±5.95
MIMAT0003477_mmu-miR-669a-5p	20.9±4	20.93±2.24
MIMAT0000160_mmu-miR-150-5p	36.84±6.08	55.95±16.27
MIMAT0004876_5_mmu-miR-466b-3p	14.99±4.96	23.42±3.64
MIMAT0016998_mmu-miR-190a-3p	26.04±3.03	12.33±2.19
MIMAT0014877_1_mmu-miR-3084-3p	31.56±1.92	13.11±1.27
MIMAT0029850_mmu-miR-7672-5p	30.75±2.51	22.47±2.36
MIMAT0031394_mmu-miR-8093	17.75±3.41	27.78±1.34
MIMAT0029807_mmu-miR-219b-3p	40.73±12.99	16.98±2.53
MIMAT0020629_mmu-miR-5121	19.27±4.48	18.71±2.33
MIMAT0001637_mmu-miR-452-5p	25.96±3.06	23.32±1.55
MIMAT0029823_mmu-miR-7658-3p	23.28±2	19.66±4.46
MIMAT0004210_mmu-miR-804	14.89±4.51	16.85±2.84
MIMAT0027843_mmu-miR-6970-3p	42.12±10.1	1.93±0.43
MIMAT0027870_mmu-miR-6984-5p	34.52±4.07	33.22±5.89
MIMAT0025077_mmu-miR-6335	24.67±1.66	19.48±1.39
MIMAT0017240_mmu-miR-802-3p	14.44±2.89	2.57±0.52
MIMAT0004618_mmu-miR-196a-2-3p	16.27±2.05	13.48±1.59
MIMAT0000210_1_mmu-miR-181a-5p	24.17±1.64	31.05±2.89
MIMAT0000532_mmu-miR-23a-3p	22.96±3.88	14.66±2.33
MIMAT0027923_mmu-miR-7009-3p	15.73±1.29	17.51±1.29
MIMAT0014825_mmu-miR-1251-3p	22.03±3.96	7.94±2.1
MIMAT0025084_mmu-miR-6341	19.57±0.76	8.99±1.21
MIMAT0028006_mmu-miR-7051-5p	22.62±4.86	5.68±0.82
MIMAT0004628_mmu-miR-21a-3p	16.63±0.93	11.17±0.8
MIMAT0017005_mmu-miR-122-3p	10.92±3.11	3.42±1.45
MIMAT0017274_mmu-miR-466h-3p	14.87±1.35	22.44±1.86
MIMAT0028394_mmu-miR-7213-5p	19.05±2.87	6.05±0.66
MIMAT0022363_mmu-miR-5618-5p	11.82±1.8	7.55±0.58
MIMAT0003892_mmu-miR-762	12.64±1.19	7.45±0.7
MIMAT0029834_mmu-miR-7664-5p	13.93±3.25	13.7±3.17
MIMAT0022374_mmu-miR-5623-3p	26.76±4.03	28.67±4.34
MIMAT0025167_mmu-miR-378d	12.36±1.59	2.66±1.02
MIMAT0020641_mmu-miR-5130	17.39±3.44	3.16±0.71
MIMAT0025172_mmu-miR-6417	14.23±1.92	22.36±4.83
MIMAT0017243_4_mmu-miR-669a-3p	14.04±1.48	11.35±1.07
MIMAT0027808_mmu-miR-6954-5p	18.07±1.36	7.14±1.26
MIMAT0017070_mmu-miR-7a-2-3p	15.17±1.38	9.85±0.93
MIMAT0017331_mmu-miR-1197-5p	13.79±3.72	24.63±6.53
MIMAT0017216_mmu-miR-487b-5p	8.14±2.08	4.51±0.68
MIMAT0027909_mmu-miR-6973b-3p	13.78±0.48	13.88±2.12
MIMAT0027944_mmu-miR-7020-5p	16.52±3.24	9.26±2.88
MIMAT0017249_mmu-miR-495-5p	14.83±1.05	21.93±2.88
MIMAT0027912_mmu-miR-7004-5p	11.97±0.54	5.71±0.79

MIMAT0003490_mmu-miR-700-3p	16.52±3.24	9.26±2.88
MIMAT0028014_mmu-miR-7055-5p	13.08±1.89	8.24±0.95
MIMAT0003469_mmu-miR-690	6.92±2.47	9.05±3.16
MIMAT0003732_mmu-miR-668-3p	24.95±4.86	11.62±0.99
MIMAT0027803_mmu-miR-6951-3p	10.34±1.41	5.89±2.07
MIMAT0005843_mmu-miR-1188-5p	10.14±1.22	10.12±1.47
MIMAT0005832_mmu-miR-669g	8.02±1.86	5±2.2
MIMAT0003151_mmu-miR-378a-3p	10.34±1.41	5.89±2.07
MIMAT0014866_mmu-miR-3079-5p	8.19±1.74	11.59±1.84
MIMAT0005837_mmu-miR-1187	21.18±3.82	22.3±3.32
MIMAT0004627_mmu-miR-20a-3p	9.7±2.39	8.13±1.5
MIMAT0017254_mmu-miR-499-3p	7.26±2.18	6.73±0.83
MIMAT0003725_mmu-miR-675-5p	10.33±0.92	7.9±0.76
MIMAT0027814_mmu-miR-6957-5p	18.89±5.18	3.91±1.57
MIMAT0028127_mmu-miR-7115-5p	11.2±1.53	11.78±1.06
MIMAT0004872_1_mmu-miR-465b-3p	6.49±1.71	6.16±0.9
MIMAT0029901_mmu-miR-290b-3p	5.68±1.42	4.77±0.88
MIMAT0009459_mmu-miR-1982-5p	7.25±1.86	9.8±1.25
MIMAT0000124_mmu-miR-15b-5p	9.07±2.13	6.56±0.33
MIMAT0003186_mmu-miR-369-3p	9.95±1.85	5.62±0.5
MIMAT0025128_mmu-miR-6382	8.11±1.8	5.35±0.5
MIMAT0004930_mmu-miR-466d-5p	10.2±1.13	6.5±0.66
MIMAT0016981_mmu-miR-99a-3p	11.28±2.99	14.05±1.99
MIMAT0029855_mmu-miR-3569-3p	9.74±0.82	0.87±0.39
MIMAT0004933_mmu-miR-878-3p	8.97±1.62	3.15±0.42
MIMAT0003376_mmu-miR-302c-3p	2.91±1.72	1.17±0.57
MIMAT0000161_mmu-miR-151-3p	8.33±1.86	3.01±0.47
MIMAT0029801_mmu-miR-7649-3p	5.09±1	5.36±1.54
MIMAT0017030_mmu-miR-328-5p	2.93±1.25	1.8±0.5
MIMAT0028091_mmu-miR-7092-3p	5.11±0.97	4.48±1.31
MIMAT0020610_mmu-miR-5103	3.72±1.02	4.88±0.67
MIMAT0014842_mmu-miR-3068-5p	9.5±0.99	6.19±1.75
MIMAT0017017_mmu-let-7f-2-3p	4.93±0.8	30.81±5.14
MIMAT0029833_mmu-miR-7663-3p	6.85±0.95	8.16±1.54
MIMAT0014874_mmu-miR-3083-5p	3.22±1.23	1.71±0.44
MIMAT0000379_mmu-miR-301a-3p	2.53±1.47	1.35±0.52
MIMAT0027940_mmu-miR-7018-5p	4.31±0.99	8.3±0.45
MIMAT0028086_mmu-miR-7090-5p	4.23±0.82	1.11±0.27
MIMAT0025161_mmu-miR-6408	6.03±0.72	7.5±0.36
MIMAT0031414_mmu-miR-1668	4.67±0.31	4.13±0.46
MIMAT0028438_mmu-miR-7235-5p	7.05±1.65	9.17±1.02
MIMAT0004746_mmu-miR-409-5p	6.54±1.52	10.48±2.19
MIMAT0004665_mmu-miR-218-1-3p	2.57±1.14	3.31±0.99
MIMAT0025148_mmu-miR-21c	4.26±1.43	4.32±0.57
MIMAT0000533_1_mmu-miR-26a-5p	2.39±1.06	4±0.74
MIMAT0027702_mmu-miR-6901-5p	7.9±1.18	8.02±0.68

MIMAT0004897_mmu-miR-654-5p	7.67±0.97	11.21±1
MIMAT0027894_mmu-miR-6996-5p	5.39±0.98	5.99±1.74
MIMAT0022378_mmu-miR-5624-3p	4.84±1.96	2.69±0.41
MIMAT0014837_mmu-miR-3065-3p	6.41±1.88	8.24±0.93
MIMAT0022355_1_mmu-miR-5615-5p	5.24±1.06	11.28±2.23
MIMAT0029867_mmu-miR-1191b-3p	6.19±1.31	2.23±0.94
MIMAT0027865_mmu-miR-6981-3p	2.06±0.9	0.4±0.26
MIMAT0027770_mmu-miR-6935-5p	5.67±1.47	7.38±1.23
MIMAT0027855_mmu-miR-6976-3p	6.91±1.07	15.69±1.57
MIMAT0025095_mmu-miR-6352	2.38±0.72	1.34±0.25
MIMAT0003477_4_mmu-miR-669a-5p	3.37±0.5	5.92±0.68
MIMAT0014897_mmu-miR-3088-5p	3.72±0.83	7.81±1.43
MIMAT0017067_mmu-miR-181b-1-3p	1.62±0.96	1.4±0.34
MIMAT0027831_mmu-miR-6965-3p	2.56±0.87	2.19±0.46
MIMAT0024861_mmu-miR-6240	2.12±0.79	2.12±0.43
MIMAT0004747_mmu-miR-411-5p	2.38±0.72	1.34±0.25
MIMAT0002108_8_mmu-miR-467a-3p	6.93±2.61	1.69±0.64
MIMAT0028038_mmu-miR-7067-5p	2.81±0.75	3.56±0.65
MIMAT0014911_mmu-miR-3095-5p	2.99±1.28	1.13±0.41
MIMAT0017172_mmu-miR-410-5p	4.36±0.89	11.51±2.07
MIMAT0003457_1_mmu-miR-680	4.93±0.88	8.11±1.33
MIMAT0029909_mmu-miR-7689-3p	3.07±0.3	0.67±0.15
MIMAT0027822_mmu-miR-6961-5p	1.31±0.7	1±0.44
MIMAT0004826_mmu-miR-146b-3p	2.16±0.69	2.65±0.65
MIMAT0004324_mmu-miR-181d-5p	2.51±0.87	0.5±0.35
MIMAT0014958_mmu-miR-1912-3p	5.14±1.15	8.61±1.9
MIMAT0000539_1_mmu-miR-92a-3p	3.13±0.15	1.84±0.36
MIMAT0027852_mmu-miR-6975-5p	3.77±1.04	2.54±0.96
MIMAT0003457_mmu-miR-680	3.97±0.87	4.76±0.62
MIMAT0028003_mmu-miR-7049-3p	2.86±0.49	2.25±0.55
MIMAT0017004_mmu-miR-206-5p	3.04±0.65	3.55±0.98
MIMAT0005851_mmu-miR-1193-3p	2.32±0.5	1.76±0.4
MIMAT0017265_mmu-miR-871-3p	3.47±0.81	1.25±0.37
MIMAT0003479_mmu-miR-669c-5p	1.11±0.78	0.09±0.09
MIMAT0028058_mmu-miR-7076-5p	6.61±1.53	6.49±2.1
MIMAT0004789_mmu-miR-450a-2-3p	2.48±1.28	3.5±0.28
MIMAT0004622_mmu-let-7c-1-3p	3.48±1.1	3.49±1.01
MIMAT0009402_mmu-miR-1938	3.11±0.38	0.79±0.45
MIMAT0020644_mmu-miR-5133	1.36±0.69	0.8±0.3
MIMAT0022366_mmu-miR-5619-3p	2.68±0.79	1.97±0.75
MIMAT0028435_mmu-miR-7233-3p	2.43±0.82	7.08±0.79
MIMAT0027777_mmu-miR-6938-3p	0.82±0.82	1.53±0.23
MIMAT0003477_11_mmu-miR-669a-5p	4.32±1.64	3.18±0.45
MIMAT0000223_mmu-miR-193a-3p	1.66±0.74	1.93±0.37
MIMAT0004871_mmu-miR-465b-5p	2.76±0.97	0.64±0.38
MIMAT0017232_mmu-miR-301b-5p	2.42±0.77	0.09±0.09

MIMAT0004851_mmu-miR-883b-3p	3.41±1.65	1.8±0.57
MIMAT0027799_mmu-miR-6949-3p	1.01±0.59	1.59±0.56
MIMAT0017353_mmu-miR-664-5p	1.53±0.67	1.08±0.32
MIMAT0003742_mmu-miR-455-3p	1.26±0.49	2.2±0.52
MIMAT0000653_mmu-miR-28a-5p	1.12±0.65	0.41±0.26
MIMAT0000582_mmu-miR-338-3p	2.51±0.55	5.26±0.7
MIMAT0022359_mmu-miR-5616-5p	0.55±0.55	0.09±0.09
MIMAT0003409_7_mmu-miR-467a-5p	1.55±0.28	0.94±0.27
MIMAT0004875_2_mmu-miR-466b-5p	1.53±0.67	0.58±0.23
MIMAT0001419_mmu-miR-433-5p	0.55±0.55	0±0
MIMAT0001094_mmu-miR-412-3p	2.95±1.16	0.86±0.34
MIMAT0027960_mmu-miR-7028-5p	0.8±0.52	0.77±0.33
MIMAT0002108_1_mmu-miR-467a-3p	3.89±0.87	4.53±0.2
MIMAT0029858_mmu-miR-7675-5p	1.57±0.31	2.1±0.52
MIMAT0029886_mmu-miR-7683-5p	1.83±0.29	0±0
MIMAT0027693_mmu-miR-6896-3p	1.09±0.45	0.5±0.23
MIMAT0000375_1_mmu-miR-297a-5p	3.65±0.75	3.21±0.73
MIMAT0025083_mmu-miR-6340	1.66±0.74	0.31±0.18
MIMAT0022383_mmu-miR-344h-5p	1.24±0.73	0.81±0.48
MIMAT0027812_mmu-miR-6956-5p	2.76±0.67	4.92±1.09
MIMAT0027785_mmu-miR-6942-3p	1.85±0.32	3.14±0.45
MIMAT0025100_mmu-miR-6357	0.78±0.52	1.31±0.65
MIMAT0001546_1_mmu-miR-450a-5p	1.94±0.98	1.47±0.51
MIMAT0027757_mmu-miR-6928-3p	3.52±1.51	1.76±0.51
MIMAT0014948_mmu-miR-3108-3p	1.09±0.45	0.5±0.23
MIMAT0027878_mmu-miR-6988-5p	1.85±0.32	3.14±0.45
MIMAT0003477_10_mmu-miR-669a-5p	5.04±1.11	2.85±0.74
MIMAT0025159_mmu-miR-6406	0.84±0.53	0.14±0.14
MIMAT0028434_mmu-miR-7233-5p	2.3±0.61	0.95±0.46
MIMAT0003464_mmu-miR-686	1.05±0.61	0±0
MIMAT0025079_mmu-miR-6336	1.09±0.45	0.09±0.09
MIMAT0027839_mmu-miR-6968-3p	0.76±0.25	0±0
MIMAT0022505_mmu-miR-5710	1.04±0.05	0.14±0.14
MIMAT0000229_mmu-miR-199a-5p	0.27±0.27	0.4±0.26
MIMAT0029862_mmu-miR-129b-5p	0.27±0.27	0.14±0.14
MIMAT0028433_mmu-miR-7232-3p	3.07±0.9	0.81±0.48
MIMAT0005849_mmu-miR-1191a	0.27±0.27	0.23±0.13
MIMAT0027928_mmu-miR-7012-5p	1.59±0.72	0.54±0.07
MIMAT0029885_mmu-miR-7682-3p	3.39±1.7	6.44±1.38
MIMAT0003893_mmu-miR-761	0.27±0.27	0±0
MIMAT0004545_mmu-miR-200b-5p	0.51±0.29	0.14±0.14
MIMAT0027344_mmu-miR-6516-3p	0.76±0.25	0.13±0.13
MIMAT0009419_mmu-miR-669m-3p	0.56±0.32	0±0
MIMAT0029831_mmu-miR-7662-3p	0.79±0.27	0.71±0.32
MIMAT0028400_mmu-miR-7216-5p	2.47±1.46	7.03±1.37
MIMAT0025130_mmu-miR-6384	0.51±0.29	0.31±0.18

MIMAT0027991_mmu-miR-7043-3p	2.16±1.24	1.16±0.42
MIMAT0014808_1_mmu-miR-344d-3p	0.27±0.27	0±0
MIMAT0025170_mmu-miR-6416-5p	0.56±0.32	0±0
MIMAT0009415_mmu-miR-1948-3p	0.74±0.45	0.9±0.1
MIMAT0000209_1_mmu-miR-129-5p	0.27±0.27	0±0
MIMAT0000588_mmu-miR-341-3p	0.27±0.27	0.09±0.09
MIMAT0017323_mmu-miR-669k-5p	0.51±0.29	0.09±0.09
MIMAT0027740_mmu-miR-6920-5p	1.86±0.57	0.67±0.15
MIMAT0029842_mmu-miR-7668-5p	0.56±0.32	0.32±0.19
MIMAT0000708_mmu-miR-363-3p	0.85±0.55	0.58±0.3
MIMAT0025178_mmu-miR-451b	0.27±0.27	1.52±0.75
MIMAT0025166_mmu-miR-6413	0.27±0.27	0±0
MIMAT0017342_mmu-miR-1943-3p	0.27±0.27	0.4±0.26
MIMAT0009455_mmu-miR-1983	1.24±0.46	0.58±0.3
MIMAT0017058_mmu-miR-26a-2-3p	0.81±0.27	0.35±0.35
MIMAT0014836_mmu-miR-3065-5p	1.92±0.97	0.41±0.26
MIMAT0027845_mmu-miR-6971-3p	0.27±0.27	0±0
MIMAT0027733_mmu-miR-6916-3p	0.76±0.25	0±0
MIMAT0027715_mmu-miR-6907-3p	0.27±0.27	1.49±0.67
MIMAT0028409_mmu-miR-7220-3p	1.25±0.57	2.68±0.78
MIMAT0007870_mmu-miR-1900	0.27±0.27	0.23±0.14
MIMAT0027789_mmu-miR-6944-3p	0.27±0.27	0.99±0.26
MIMAT0028021_mmu-miR-7058-3p	0.51±0.29	0.4±0.15
MIMAT0014949_mmu-miR-3109-5p	2.06±0.4	1.62±0.23
MIMAT0002106_mmu-miR-465a-5p	0.79±0.27	1.75±0.26
MIMAT0000590_mmu-miR-342-3p	1.26±0.63	1.03±0.22
MIMAT0029830_mmu-miR-7662-5p	0.27±0.27	0±0
MIMAT0000521_mmu-let-7a-5p	0.27±0.27	0.13±0.13
MIMAT0004931_mmu-miR-466d-3p	0.81±0.27	0.28±0.28
MIMAT0036459_mmu-miR-9768-5p	0.56±0.32	0.54±0.07
MIMAT0004524_mmu-miR-30b-3p	0.27±0.27	0.09±0.09
MIMAT0000220_mmu-miR-190a-5p	2.56±0.85	1.59±0.91
MIMAT0028421_mmu-miR-7226-3p	0.85±0.55	0.41±0.26
MIMAT0017036_mmu-miR-148b-5p	1.86±0.57	1.08±0.66
MIMAT0005845_mmu-miR-466k	0.27±0.27	0.36±0.26
MIMAT0029799_mmu-miR-7648-3p	1.24±0.46	2.29±0.41
MIMAT0027980_mmu-miR-7038-5p	1.36±0.73	0.77±0.33
MIMAT0028060_mmu-miR-7077-5p	0.27±0.27	0±0
MIMAT0000740_mmu-miR-376a-3p	0.27±0.27	0±0
MIMAT0027993_mmu-miR-7044-3p	1.74±0.85	0.14±0.14
MIMAT0028425_mmu-miR-7228-3p	0.76±0.25	1.22±0.2
MIMAT0027969_mmu-miR-7032-3p	0.79±0.27	0.4±0.15
MIMAT0003895_mmu-miR-764-3p	0.27±0.27	0.54±0.23
MIMAT0027776_mmu-miR-6938-5p	0.97±0.65	0.44±0.15
MIMAT0029881_mmu-miR-465d-3p	2.67±1.04	1.36±0.64
MIMAT0027788_mmu-miR-6944-5p	0.74±0.45	1.98±0.47

MIMAT0000127_mmu-miR-29b-3p	0.74±0.45	1.3±0.12
MIMAT0014933_mmu-miR-3102-5p	0.27±0.27	0±0
MIMAT0004857_mmu-miR-147-3p	1.22±0.57	1.62±0.24
MIMAT0031407_mmu-miR-8103	0.85±0.55	1±0.58
MIMAT0003898_mmu-miR-760-3p	0.27±0.27	0.13±0.13
MIMAT0017071_mmu-miR-7b-3p	0.27±0.27	0±0
MIMAT0029811_mmu-miR-7652-3p	0.51±0.29	0.31±0.18
MIMAT0031400_mmu-miR-8098	0.79±0.27	0.23±0.13
MIMAT0027848_mmu-miR-6973a-5p	0.27±0.27	0.09±0.09
MIMAT0029832_mmu-miR-7663-5p	1.02±0.38	0.55±0.23
MIMAT0027749_mmu-miR-6924-3p	0.27±0.27	0±0
MIMAT0000678_mmu-miR-7b-5p	0.53±0.3	0.13±0.13
MIMAT0031421_mmu-miR-8115	0.51±0.29	0.37±0.37
MIMAT0029828_mmu-miR-7661-5p	0.76±0.25	0.09±0.09
MIMAT0000149_mmu-miR-137-3p	0.81±0.27	0.67±0.34
MIMAT0028031_mmu-miR-7063-3p	0.53±0.3	0.98±0.44
MIMAT0014856_mmu-miR-3074-5p	3.06±0.66	0.99±0.58
MIMAT0004636_mmu-miR-93-3p	0.56±0.32	0±0
MIMAT0027775_mmu-miR-6937-3p	0.53±0.3	0.13±0.13
MIMAT0004877_1_mmu-miR-466c-5p	0.27±0.27	0.09±0.09
MIMAT0014832_mmu-miR-3063-5p	0.27±0.27	0±0
MIMAT0031393_mmu-miR-8092	0.27±0.27	0.54±0.38
MIMAT0000652_mmu-miR-25-3p	1.36±0.73	0.77±0.33
MIMAT0000387_mmu-miR-130b-3p	0.27±0.27	0±0
MIMAT0017081_mmu-miR-381-5p	2.52±0.55	3.27±0.58
MIMAT0009436_mmu-miR-1963	0.56±0.32	0.18±0.18
MIMAT0028136_mmu-miR-7119-3p	3.66±1.2	1.08±0.55
MIMAT0027936_mmu-miR-7016-5p	0.56±0.32	0.54±0.07
MIMAT0000743_mmu-miR-379-5p	0.27±0.27	0.09±0.09
MIMAT0009422_mmu-miR-1951	0.51±0.29	0.32±0.19
MIMAT0027896_mmu-miR-6997-5p	0.53±0.3	0±0
MIMAT0027964_mmu-miR-7030-5p	2.28±0.93	6.17±0.91
MIMAT0014922_mmu-miR-3101-3p	0.81±0.27	0.14±0.14
MIMAT0004898_mmu-miR-654-3p	2.67±1.04	1.36±0.64
MIMAT0001090_mmu-miR-409-3p	0.74±0.45	0.35±0.35
MIMAT0022386_mmu-miR-5627-3p	1.71±1	3.31±0.72

CPM: raw counts per million

Supplementary Table 2 Lists of the miRNA in milk and pup serum significant affected by maternal HFD.

The miRNA in milk

miRNA name	log2 FC (CON/HFD)	P - value	FDR
MIMAT0020629_mmu-miR-5121	-2.3	0.000393515	0.006895066
MIMAT0000528_mmu-miR-18a-5p	-1.8	3.88E-05	0.00094492
MIMAT0024861_mmu-miR-6240	-1.7	1.07E-05	0.000565426
MIMAT0020637_mmu-miR-5126	-1.7	0.000114592	0.00550042
MIMAT0000223_mmu-miR-193a-3p	-1.1	2.28E-05	0.000655548
MIMAT0000513_mmu-miR-19b-3p	-0.8	2.63E-06	8.85E-05
MIMAT0000584_mmu-miR-339-5p	-0.8	0.002654441	0.036428206
MIMAT0000545_mmu-miR-98-5p	-0.7	0.000496202	0.008332056
MIMAT0000653_mmu-miR-28a-5p	-0.7	4.94E-05	0.001047009
MIMAT0019345_mmu-miR-1843b-5p	-0.7	3.61E-05	0.00094492
MIMAT0000162_mmu-miR-152-3p	-0.6	0.000146914	0.002691195
MIMAT0004617_mmu-miR-148a-5p	0.5	0.00060173	0.009699892
MIMAT0005443_mmu-miR-181a-2-3p	0.6	0.000124837	0.002395689
MIMAT0004529_mmu-miR-125b-2-3p	0.8	9.57E-05	0.001928765
MIMAT0000208_mmu-miR-10b-5p	0.9	1.71E-06	6.27E-05
MIMAT0004580_mmu-miR-34c-3p	1	0.002825465	0.036731049
MIMAT0004628_mmu-miR-21a-3p	1	0.002084915	0.030007888
MIMAT0003509_mmu-miR-501-3p	1.2	0.0016694	0.024917335
MIMAT0000230_mmu-miR-199a-3p	1.3	0.001014802	0.015729435
MIMAT0000155_mmu-miR-142a-3p	1.6	4.20E-05	0.00094492
MIMAT0031402_mmu-miR-142b	1.6	4.22E-05	0.00094492
MIMAT0017068_mmu-miR-181c-3p	1.7	1.03E-13	8.30E-12
MIMAT0017078_mmu-miR-375-5p	2.7	9.58E-08	3.86E-06

The miRNA in pup serum

miRNA name	log2 FC(CON/HFD)	P - value	FDR
MIMAT0014809_mmu-miR-1298-5p	-2.2	0.02433	0.89819
MIMAT0000212_mmu-miR-183-5p	-1.9	3.29E-12	2.18E-09
MIMAT0004746_mmu-miR-409-5p	-1.8	0.009243	0.611896
MIMAT0009426_mmu-miR-1955-5p	-1.7	0.009965	0.611896
MIMAT0017060_mmu-miR-221-5p	-1.6	0.000504	0.111737
MIMAT0016997_mmu-miR-187-5p	-1.5	0.046999	1
MIMAT0003509_mmu-miR-501-3p	-1.2	1.34E-08	5.93E-06
MIMAT0016996_mmu-miR-185-3p	-0.9	0.048505	1
MIMAT0031410_mmu-miR-3535	-0.9	0.021044	0.89819
MIMAT0004842_mmu-miR-879-5p	-0.8	0.002836	0.401793
MIMAT0000214_mmu-miR-185-5p	-0.8	0.031667	1
MIMAT0000655_mmu-miR-100-5p	-0.8	9.63E-08	3.20E-05
MIMAT0000131_mmu-miR-99a-5p	-0.7	1.50E-12	1.99E-09
MIMAT0004661_mmu-miR-28a-3p	-0.7	2.38E-07	6.33E-05

MIMAT0004862_mmu-miR-877-3p	-0.7	0.043928	1
MIMAT0000233_mmu-miR-200b-3p	-0.6	0.000815	0.154652
MIMAT0000517_mmu-miR-192-5p	-0.3	0.036872	1
MIMAT0000533_mmu-miR-26a-5p	-0.3	0.011191	0.646634
MIMAT0000580_mmu-miR-148b-3p	-0.3	0.019681	0.888197
MIMAT0000533_1_mmu-miR-26a-5p	-0.3	0.010129	0.611896
MIMAT0000221_mmu-miR-191-5p	-0.3	0.003023	0.401793
MIMAT0019345_mmu-miR-1843b-5p	-0.2	0.03637	1
MIMAT0000140_1_mmu-miR-128-3p	-0.2	0.012108	0.665459
MIMAT0000161_mmu-miR-151-3p	-0.2	0.008496	0.611896
MIMAT0000130_mmu-miR-30b-5p	-0.1	0.045445	1
MIMAT0000652_mmu-miR-25-3p	0.2	0.024228	0.89819
MIMAT0000238_mmu-miR-205-5p	0.2	0.033291	1
MIMAT0000540_mmu-miR-93-5p	0.2	0.001335	0.221848
MIMAT0000146_mmu-miR-134-5p	0.3	0.022538	0.89819
MIMAT0000545_mmu-miR-98-5p	0.3	0.027914	1
MIMAT0000766_mmu-miR-335-5p	0.3	0.00951	0.611896
MIMAT0003732_mmu-miR-668-3p	0.3	0.033255	1
MIMAT0001421_mmu-miR-434-5p	0.3	0.02005	0.888197
MIMAT0000160_mmu-miR-150-5p	0.3	0.009	0.611896
MIMAT0001091_mmu-miR-410-3p	0.3	0.016037	0.787846
MIMAT0001420_mmu-miR-433-3p	0.4	0.016599	0.787846
MIMAT0000157_mmu-miR-145a-5p	0.4	0.014023	0.716789
MIMAT0003120_mmu-miR-483-3p	0.4	0.012518	0.665459
MIMAT0001418_mmu-miR-431-5p	0.4	0.007138	0.611896
MIMAT0004530_mmu-miR-127-5p	0.4	0.006872	0.611896
MIMAT0003453_mmu-miR-497a-5p	0.5	0.006365	0.611896
MIMAT0000124_mmu-miR-15b-5p	0.5	0.005789	0.611896
MIMAT0000769_mmu-miR-133b-3p	0.5	0.024209	0.89819
MIMAT0003734_mmu-miR-667-3p	0.5	0.006367	0.611896
MIMAT0004782_mmu-miR-483-5p	0.5	0.046625	1
MIMAT0017018_mmu-miR-16-2-3p	0.6	0.039794	1
MIMAT0011213_mmu-miR-2137	0.7	0.035012	1
MIMAT0003473_1_mmu-miR-133a-5p	0.7	0.047528	1
MIMAT0000567_mmu-miR-329-3p	1	0.023432	0.89819
MIMAT0004519_mmu-let-7g-3p	1.4	0.046664	1
MIMAT0004539_mmu-miR-183-3p	1.5	0.007954	0.611896

Supplementary Table 3 Statistic summary

Figure 1A-AG

Up or down in high fat diet mice compared with low fat diet mice

	Diet effect		Day effect		Interaction	
	P Value	Change	P Value	Change	P Value	Change
**Figure 1A**	0.024	up	<0.0001	up	<0.0001	yes
**Figure 1B**	0.673	no	<0.0001	up	>0.9999	no
**Figure 1C**	0.0067	up	<0.0001	up	<0.0001	yes
**Figure 1D**	0.5997	no	<0.0001	up	0.9885	no
**Figure 1E**	0.04	up	<0.0001	up	<0.0001	yes
**Figure 1F**	0.0013	up	<0.0001	up	<0.0001	yes
**Figure 1G**	0.0163	up	<0.0001	up	<0.0001	yes
**Figure 1H**	0.0011	up	<0.0001	up	<0.0001	yes

Figure 1I-1J

Area	Subcutaneous fat		Visceral fat		Total fat	
	P	change	P	change	P	change
**Figure 1I male**	0.04	up	0.5512	no	0.0612	no
**Figure 1I female**	0.0122	up	0.1323	no	0.0167	up
**Figure 1J male**	0.04	up	0.0057	up	0.0289	up
**Figure 1J female**	0.0047	up	0.029	up	0.0061	up

Figure 2B

Up or down in high fat diet mice compared with low fat diet mice

Name	Male		Female	
	P Value	Change	P Value	Change
**AGRP**	0.083581	no	0.793746	no
**NPY**	0.55128	no	0.006104	down
**NPY1R**	0.354483	no	0.0108	down
**NPY2R**	0.152947	no	0.054585	no
**NPY5R**	0.433106	no	0.030023	down
**POMC**	0.003447	up	0.929845	no
**CARTPT**	0.000137	up	0.014008	up
**MC4R**	0.299701	no	0.096373	no
**HTR1A**	0.023426	down	0.0019	down
**HTR1B**	0.000338	down	1.48E-08	down
**HTR1D**	0.602519	no	0.013238	down
**HTR2A**	0.000291	down	0.00049	down
**HTR2C**	0.003436	down	9.85E-05	down
**HTR3A**	0.017096	dpwn	0.895978	no
**HTR4**	0.550919	no	0.028467	down

**HTR5A**	0.189574	no	0.001146	down
**HTR5B**	0.178117	no	0.490357	no
**HTR6**	0.424353	no	0.007722	down
**OPRD1**	0.417739	no	0.019075	down
**OPRK1**	0.199407	no	0.000101	down
**OPRM1**	0.372988	no	0.031992	down
**DRD1**	0.57586	no	0.022982	down
**DRD2**	0.346635	no	0.002128	down
**DRD5**	0.061185	no	0.000665	down

Figure 3A-3F and Figure 33M-3R

Up or down in high fat diet mice compared with low fat diet mice

**Area**	**ME**		**ARC**		**VMH**		**DMH**	
	P	change	P	change	P	change	P	change
**Figure 3A**	0.8513	no	0.0078	down	0.1139	no	0.1509	no
**Figure 3B**	0.582	no	0.2999	no	0.5259	no	0.4814	no
**Figure 3C**	0.0358	down	<0.0001	down	0.0013	down	0.0358	down
**Figure 3D-NeuN**	0.0161	down	<0.0001	down	0.0012	down	0.0021	down
**Figure 3D-NeuN+BrdU**	0.0021	down	0.3041	no	0.8991	no	0.0012	down
**Figure 3E-NeuN**	0.2705	no	0.0563	no	0.0085	down	0.0104	down
**Figure 3E-NeuN+BrdU**	0.0003	down	0.0068	down	0.0002	down	0.004	down
**Figure 3F-NeuN**	0.006	down	0.0128	down	0.002	down	0.0014	down
**Figure 3F-NeuN+BrdU**	0.0012	down	0.0075	down	0.0006	down	<0.0001	down
**Figure 3M**	0.0109	down	0.046	down	0.8716	no	0.6339	no
**Figure 3N**	0.0255	dwon	0.0023	down	0.0554	no	0.0028	down
**Figure 3O**	0.6968	no	0.3307	no.	0.0013	down	0.0439	down
**Figure 3P-NeuN**	0.0504	no	0.0028	down	0.0007	down	0.0002	down
**Figure 3P-NeuN+BrdU**	0.2187	no	0.1996	no	0.0453	down	0.0361	down
**Figure 3Q-NeuN**	0.8004	no	0.0001	down	<0.0001	down	0.4872	no
**Figure 3Q-NeuN+BrdU**	0.0005	down	0.0036	down	0.0007	down	0.0002	down
**Figure 3R-NeuN**	0.7064	no	0.0598	no	0.1339	no	0.0165	down
**Figure 3R-NeuN+BrdU**	<0.0001	down	0.0776	no	0.0519	no	0.0055	down

Figure 3G-3Land Figure 3S-3Y

Up or down in high fat diet mice compared with low fat diet mice

**Area**	**NPY or POMC cells**		**NPY+BrdU or POMC+BrdU**	
	P	change	P	change
**Figure 3G**	0.04	up	0.2338	no
**Figure 3H**	0.3802	no	0.0106	up
**Figure 3I**	0.0479	up	0.1674	no

**Figure 3J**	0.0168	down	0.0078	down
**Figure 3K**	0.0002	down	<0.0001	down
**Figure 3L**	0.0027	down	0.0174	down
**Figure 3S**	0.0208	up	0.0164	up
**Figure 3T**	0.0123	up	0.0214	up
**Figure 3U**	0.3251	no	0.1825	no
**Figure 3V**	0.2747	no	0.142	no
**Figure 3W**	0.0004	down	<0.0001	down
**Figure 3X**	0.0006	down	0.0086	down

**Area**	**D9**		**D15**		**D21**	
	P	change	P	change	P	change
**Figure 3Y male**	0.0273	up	0.0003	up	0.0361	up
**Figure 3Y female**	0.0285	up	0.0115	up	0.1232	no

Figure 4A-4F

Up or down in high fat diet mice compared with low fat diet mice

**Area**	**ME**		**ARC**		**VMH**		**DMH**	
	P	change	P	change	P	change	P	change
**Figure 4A**	0.6985	no	0.8904	no	0.3442	no	0.6279	no
**Figure 4B**	0.0022	down	0.0107	down	0.0387	down	0.0456	down
**Figure 4C**	0.0024	down	0.0028	down	0.0008	down	0.0021	down
**Figure 4D**	0.8299	no	0.876	no	0.438	no	0.974	no
**Figure 4E**	0.0203	down	0.0372	down	0.0127	down	0.252	no
**Figure 4F**	0.1132	no	0.1848	no	0.1042	no	0.3881	no

Figure 4G

**Name**	**Male**		**Female**	
	P Value	Change	P Value	Change
**Mki67**	0.00355512	down	0.0017	down
**Neurod1**	0.00803575	down	0.7227	no
**Neurod2**	0.0027723	down	0.7939	no
**Neurod6**	0.03225876	down	0.6844	no

Figure 5A-5O and Figure 5S-5AH

Area	BrdU cells		NeuN+BrdU	
	P	change	P	change
**Figure 5A**	0.0226	down	0.0233	down
**Figure 5B**	0.0411	down	0.0387	down
**Figure 5C**	0.2475	no	0.2251	no
**Figure 5S**	0.0031	down	0.0047	down
**Figure 5T**	0.04	down	0.0382	down
**Figure 5U**	0.7399	no	0.7042	no
	**BrdU cells**		**NPY+BrdU or POMC+BrdU**	
**Figure 5D**	0.0353	down	0.3089	no
**Figure 5E**	0.0449	down	0.3329	no
**Figure 5F**	0.8015	no	0.0123	up
**Figure 5V**	0.0111	down	0.8831	no
**Figure 5W**	0.0012	down	0.1915	no
**Figure 5X**	0.1092	no	0.0311	up
**Figure 5G**	0.0096	down	0.0011	down
**Figure 5H**	0.005	down	0.0057	down
**Figure 5I**	0.1406	no	0.0091	down
**Figure 5Y**	<0.0001	down	0.0718	no
**Figure 5Z**	0.0006	down	0.0688	no
**Figure 5AB**	0.0712	no	0.0157	down
	**BrdU cells**		**GFAP+BrdU or Iba1+BrdU**	
**Figure 5J**	0.0482	down	0.0944	no
**Figure 5K**	0.0299	dwon	0.721	no
**Figure 5L**	0.1168	no	0.5185	no
**Figure 5AC**	0.0059	down	0.6779	no
**Figure 5AD**	0.0294	dowm	0.3118	no
**Figure 5AE**	0.0033	down	0.5324	no
**Figure 5M**	0.0055	down	0.089	no
**Figure 5N**	0.0237	down	>0.9999	no
**Figure 5O**	0.0551	down	0.49	no
**Figure 5AF**	0.0076	down	0.1583	no
**Figure 5AG**	0.0053	down	0.647	no
**Figure 5AH**	0.155	no	0.3739	no

Figure 5P-5R and
Figure 5AI-5AK

Area	NeuN+BrdU/BrdU cells		NPY+BrdU/BrdU cells		POMC+BrdU/BrdU cells		GFAP+BrdU/BrdU cells		Iba1+BrdU/BrdU cells	
	P	change	P	change	P	change	P	change	P	change
**Figure 5P**	0.223	no	0.1006	no	0.0796	no	0.0041	up	0.684	no
**Figure 5Q**	0.114	no	0.0014	up	0.1784	no	0.1991	no	0.465	no
**Figure 5R**	0.5577	no	0.0003	up	0.0049	down	0.271	no	0.4387	no
**Figure 5AI**	0.502	no	0.0096	up	0.9204	no	0.0045	up	0.186	no
**Figure 5AJ**	0.2949	no	0.5796	no	0.4771	no	0.1297	no	0.417	no
**Figure 5AK**	0.7414	no	0.004	up	0.038	down	0.06223	no	0.3739	no

Figure AL

Area	D9		D15		D21	
	P	change	P	change	P	change
**Figure 5AL male**	0.0002	up	0.04	up	0.04	up
**Figure 5AL female**	0.3533	up	0.415	up	0.0022	up

Up or down in high fat diet mice
compared with low fat diet mice

Figure 6A

	Pearson Correlation		
	P	r	change
**CON serum-skim**	<0.0001	0.71	yes
**CON serum-fat**	<0.0001	0.7152	yes
**HFD serum-skim**	<0.0001	0.7082	yes
**HFD serum-fat**	<0.0001	0.7049	yes

Figure 6B

	Protein		Carbohydrate		Fatty acid	
	P	change	P	change	P	change
**Figure 6B**	0.5468	no	0.4685	no	0.0001	up

Up or down in HFD mice compared with low fat diet mice

Figure 6C, 6D and 6E

Name	Diet		Milk		Brain-M		Brain-F	
	P Value	Change	P Value	Change	P Value	Change	P Value	Change
**CE**	0.0336	up	<0.0001	up	0.1197	no	0.0875	no
**CHO**	0.0005	up	0.0381	up	0.0554	no	0.6895	no
**TAG**	0.0025	up	0.0065	down	0.4985	no	0.3258	no
**DAG**	0.7121	no	0.0722	no	0.6856	no	0.0397	up
**SM**	0.9925	no	0.0014	up	0.3517	no	0.5564	no
**Cer**	0.743	no	0.0287	up	0.0049	down	0.9654	no
**GluCer**	0.908	no	0.0774	no	0.0043	down	0.8268	no
**GM3**	0.958	no	0.3048	no	0.0091	down	0.2884	no
**LBPA**	0.2711	no	0.0012	up	0.2437	no	0.2933	no
**LPC**	0.0037	down	0.0305	up	0.2055	no	0.5476	no
**PC**	0.7825	no	0.0005	up	0.2171	no	0.6427	no
**PE**	0.4449	no	0.0058	up	0.2289	no	0.582	no
**PA**	0.0754	no	0.0631	up	0.3354	no	0.543	no
**PG**	0.7064	no	0.0026	up	0.036	up	0.0042	down
**PI**	0.641	no	0.004	up	0.0237	down	0.5326	no
**PS**	0.9948	no	0.0157	up	0.0381	down	0.5899	no
**SL**					0.4666	no	0.6037	no
**Sph d18:1**					0.8637	no	0.6756	no

Figure 6G

Name	Brain-M		Brain-F	
	P Value	Change	P Value	Change
Cer d18:0/18:0	0.6115	no	0.67	no
Cer d18:0/20:0	0.1665	no	0.1072	no
Cer d18:0/22:0	0.0716	no	0.2895	no
Cer d18:1/16:0	0.4685	no	0.939	no
Cer d18:1/18:0	0.3941	no	0.5115	no
Cer d18:1/20:0	0.1749	no	0.9521	no
Cer d18:1/22:0	0.0345	down	0.9187	no
Cer d18:1/24:0	0.0357	down	0.2122	no
Cer d18:1/24:1	0.1249	no	0.6506	no
GalCer d18:0/18:0	0.0108	down	0.145	no
GalCer d18:0/22:0	0.0005	down	0.7557	no
GalCer d18:0/24:0	0.0029	down	0.6658	no
GalCer d18:0/24:1	0.0038	down	0.7689	no
GalCer d18:1/16:0	0.004	down	0.3752	no
GalCer d18:1/18:0	0.001	down	0.5814	no
GalCer d18:1/20:0	0.0039	down	0.818	no
GalCer d18:1/22:0	0.001	down	0.9534	no
GalCer d18:1/24:0	0.0211	down	0.9057	no
GalCer d18:1/24:1	0.0401	down	0.902	no
GluCer d18:0/18:0	0.0948	no	0.3799	no
GluCer d18:0/22:0	0.0011	down	0.8011	no
GluCer d18:0/24:0	0.009	down	0.4446	no
GluCer d18:0/24:1	0.0049	down	0.6158	no
GluCer d18:1/16:0	0.0101	down	0.17	no
GluCer d18:1/18:0	0.0051	down	0.1734	no
GluCer d18:1/20:0	0.0064	down	0.0964	no
GluCer d18:1/22:0	<0.0001	down	0.0992	no
GluCer d18:1/24:0	0.0263	down	0.734	no
GluCer d18:1/24:1	0.0378	down	0.9615	no

Figure 6H-milk

Name	Milk	
	P Value	Change
PG32:1	0.0372	down
PG32:2	0.3709	no
PG34:1	0.6236	no
PG34:2	0.0682	no
PG34:3	0.3413	no
PG36:1	0.0004	up
PG36:2	0.0002	up
PG36:3	0.005	up
PG36:4	0.0021	up
PI 34:1	0.8	no

**PI 34:2**	0.928	no
**PI 36:1**	0.0023	up
**PI 36:2**	0.0017	up
**PI 36:3**	0.0387	up
**PI 36:4**	0.5055	no
**PI 38:3**	0.0199	up
**PI 38:4**	0.0008	up
**PI 38:5**	0.0236	up
**PI 40:4**	0.2632	no
**PI 40:5**	0.6327	no
**PI 40:6**	0.3303	no
**PS 34:1**	0.0004	down
**PS 34:2**	0.1705	no
**PS 36:1**	0.0142	up
**PS 36:2**	0.0094	up
**PS 38:3**	0.091	no
**PS 38:4**	0.0036	up
**PS 38:5**	0.5419	no
**PS 40:4**	0.511	no
**PS 40:5**	0.0006	up
**PS 40:6**	0.0001	up
**PS 40:7**	0.0453	up

Figure 6H-brain

**Name**	**Brain-M**		**Brain-F**	
	P Value	Change	P Value	Change
**PG32:1**	0.1306	no	0.012	down
**PG32:2**	0.0117	up	0.0001	down
**PG34:1**	0.157	no	0.002	down
**PG34:2**	0.0236	up	0.003	down
**PG34:3**	0.0013	up	0.0042	down
**PG36:1**	0.1486	no	0.0627	no
**PG36:2**	0.0022	up	0.492	no
**PG36:3**	0.0044	up	0.0538	no
**PG36:4**	0.0694	no	0.6052	no
**PG38:3**	0.0064	up	0.3582	no
**PG38:4**	0.0949	no	0.2452	no
**PG38:5**	0.0152	up	0.0483	down
**PG38:6**	0.0045	up	0.2855	no
**PI 34:1**	0.0616	no	0.9981	no
**PI 34:2**	0.1215	no	0.5723	no
**PI 36:1**	0.0253	down	0.0853	no
**PI 36:2**	0.0443	down	0.909	no
**PI 36:3**	0.0714	no	0.2668	no
**PI 36:4**	0.1448	no	0.7944	no
**PI 38:3**	0.0279	down	0.2546	no

**PI 38:4**	0.0236	down	0.5225	no
**PI 38:5**	0.0047	down	0.6763	no
**PI 40:3**	0.1065	no	0.6272	no
**PI 40:4**	0.0165	down	0.2265	no
**PI 40:5**	0.0697	no	0.5961	no
**PI 40:6**	0.2337	no	0.26	no
**PS 34:1**	0.9543	no	0.6471	no
**PS 34:2**	0.2469	no	0.0973	no
**PS 36:1**	0.0018	down	0.6704	no
**PS 36:2**	0.1134	no	0.0703	no
**PS 38:3**	0.0298	down	0.455	no
**PS 38:4**	0.0291	down	0.4924	no
**PS 38:5**	0.0089	down	0.7683	no
**PS 40:4**	0.9688	no	0.878	no
**PS 40:5**	0.0032	down	0.0064	down
**PS 40:6**	0.2	no	0.4818	no
**PS 40:7**	0.3823	no	0.4234	no

Supplementary Figure 1

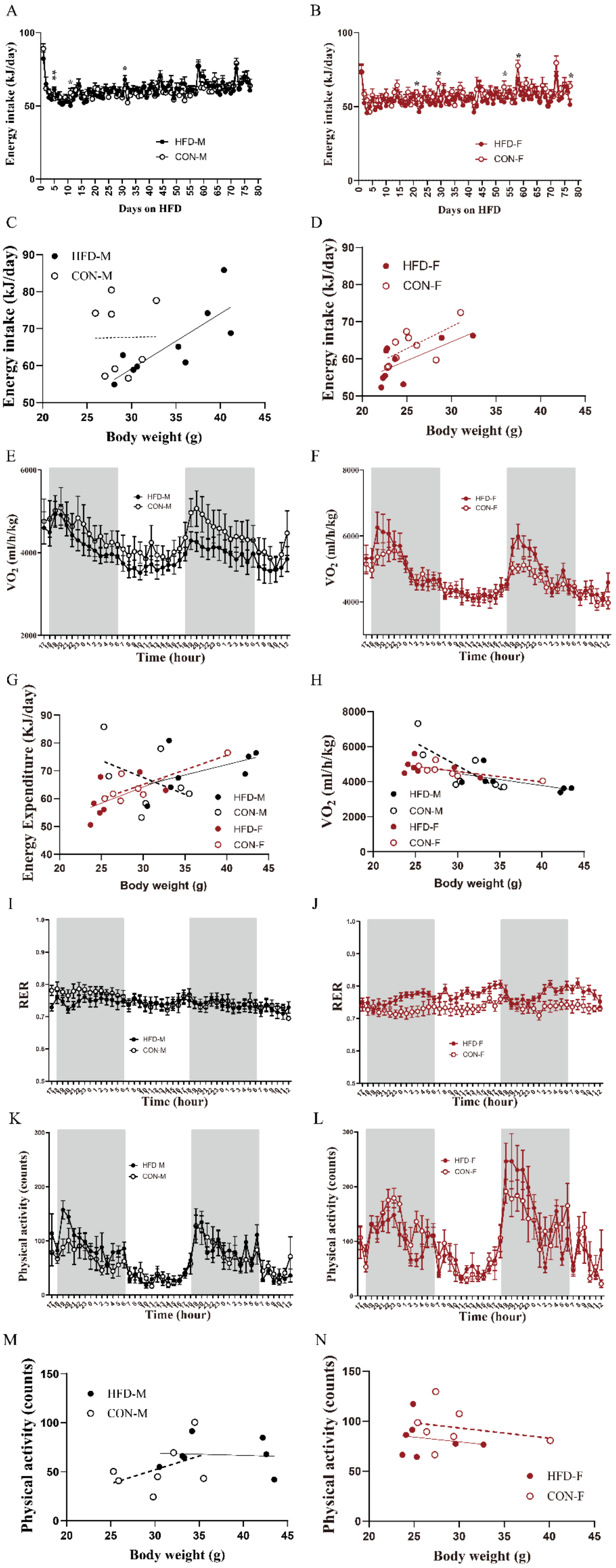

Supplementary Figure 2

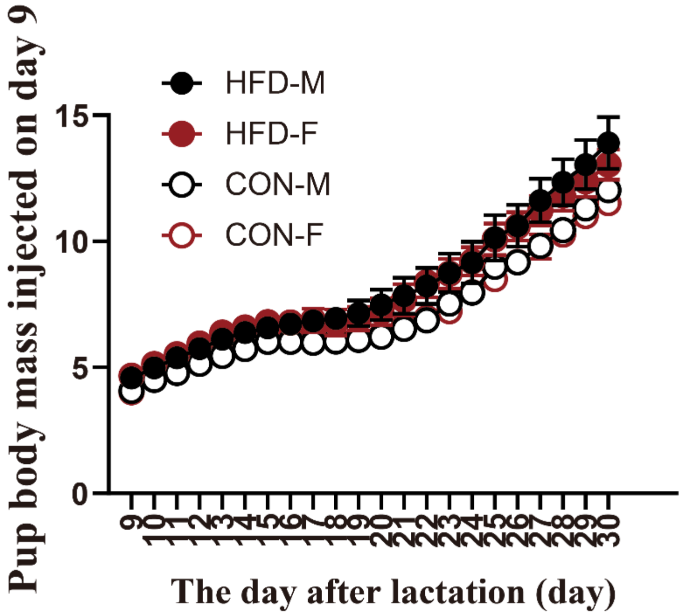

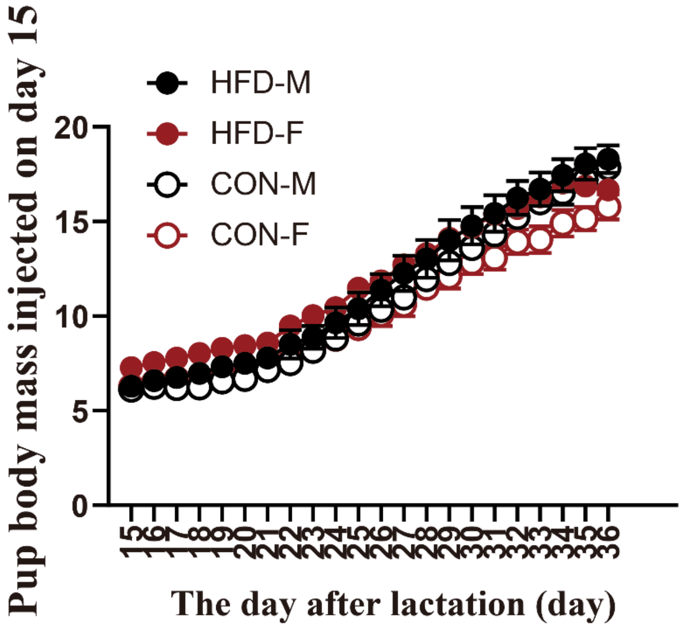

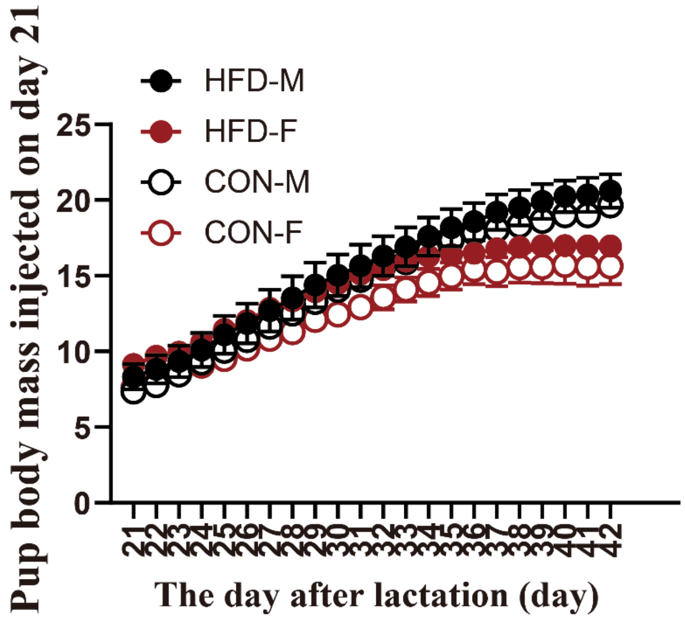

Supplementary Figure 3

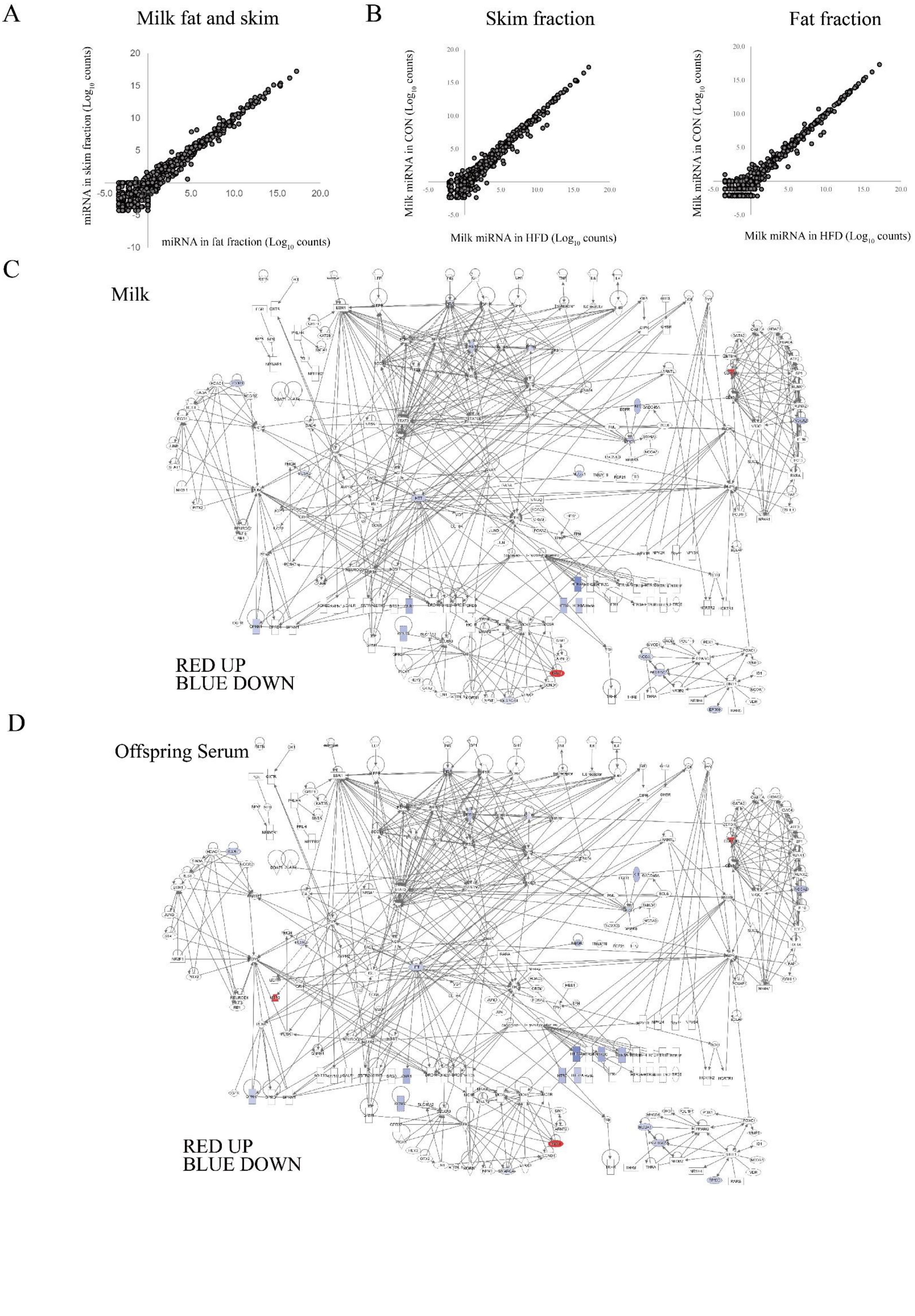

Supplementary Figure 4

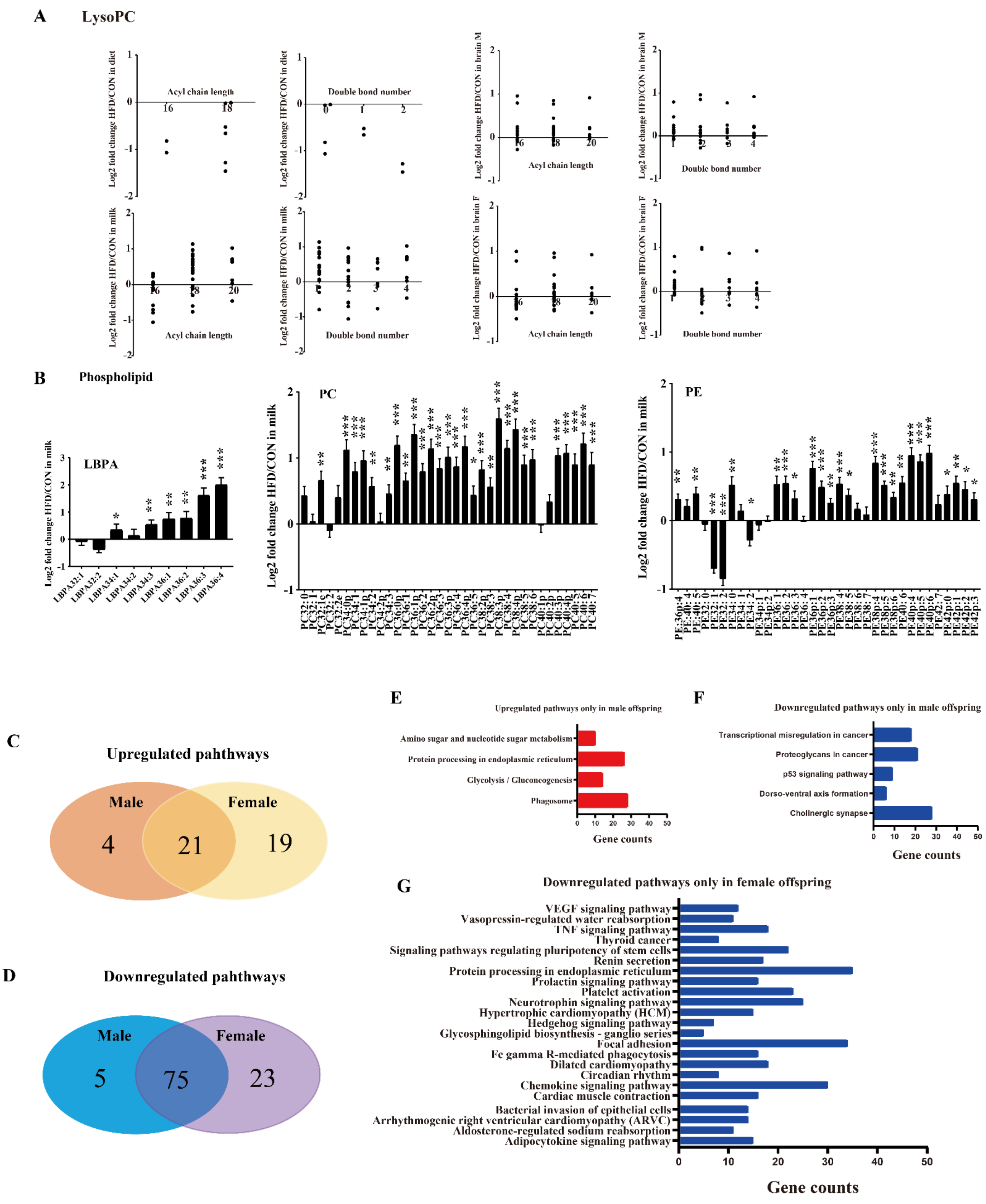

Figure S1. The effect of maternal HFD during lactation on energy balance and metabolism of adult male and female offspring mice exposed to an HFD
(A and B) Energy intake of adult male and female offspring mice that had previously been raised by mothers fed a HFD or a CON during lactation, in response to a 11-week HFD exposure in later life (n = 8-9).
(C and D) The correlation between body weight and energy intake in adult male (C) and female (D) offspring (n=8-9).
(E and F) Metabolic chamber measurements of oxygen consumption (VO2) in adult male (E) and female (F) offspring after 10 weeks of HFD exposure (n = 7).
(G and H) The correlation between body weight and energy expenditure (G) or VO2 (H) in adult male and female offspring (n = 7).
(I-L) Metabolic chamber measurements of RER (I and J) and physical activity (K and L) in male and female offspring (n = 7).
(M and N) The correlation between body weight and physical activity in adult male (M) and female (N) offspring (n = 7).
Values are represented as mean ± SEM, and analyzed by two-way ANOVA.

Figure S2. The effect of maternal HFD on the change of male and female pup's body mass, which was measured for 21 days after BrdU injection on postnatal day 9, day 15, and day 21 (n = 7). Values are represented as mean ± SEM, and analyzed by two-way ANOVA

Figure S3. Target gene networks of miRNAs in milk and pup serum in response to maternal HFD feeding during lactation
(A) The correlation of miRNAs in milk skim fraction and fat fraction (n = 8).
(B) The effect of maternal HFD on miRNAs in skim fraction (left) and fat fraction (right) (n = 8)
(C and D) Pathway diagram of target genes of miRNAs in milk (C) (n = 8) and offspring serum (D) (n = 24). Red indicates the upregulation, blue indicates the downregulation ($p < 0.05$), while gray indicates no significance. Intensity of the color is related to the absolute values of log10 (p value).

Figure S4. Lipidomics profiling in response to maternal HFD feeding during lactation
(A) LysoPC fold change (HFD versus CON) in diet (n = 2), milk (n = 8), and male and female pup hypothalami (n = 7).
(B) Phospholipids fold change including LBPA, PC, and PE (HFD versus CON) in milk (n = 8).
(C and D) Upregulated (C) and downregulated (D) pathways in male and female pup hypothalami (n = 5).
(E) KEGG pathway analysis showed upregulated pathways that are only enriched in male pup hypothalami (n = 5).
(F) KEGG pathway analysis showed downregulated pathways that are only enriched in male pup hypothalami (n = 5).
(G) KEGG pathway analysis showed downregulated pathways that are only enriched in female pup hypothalami (n = 5).
Values are represented as mean ± SEM and analyzed by unpaired Student's t test. Differences with $p < 0.05$ were considered significant. *$p < 0.05$, **$p < 0.01$, *** $p< 0.001$.
